# Glucosinolates in Human Health: Metabolic Pathways, Bioavailability, and Potential in Chronic Disease Prevention

**DOI:** 10.3390/foods14060912

**Published:** 2025-03-07

**Authors:** Sara Baldelli, Mauro Lombardo, Alfonsina D’Amato, Sercan Karav, Gianluca Tripodi, Gilda Aiello

**Affiliations:** 1 Department for the Promotion of Human Science and Quality of Life, San Raffaele Open University, Via di Val Cannuta, 247, 00166 Rome, Italy; sara.baldelli@uniroma5.it (S.B.); mauro.lombardo@uniroma5.it (M.L.); gilda.aiello@uniroma5.it (G.A.); 2 IRCCS San Raffaele Roma, 00166 Rome, Italy; 3 Department of Pharmaceutical Sciences, University of Milan, Via L. Mangiagalli 25, 20133 Milan, Italy; alfonsina.damato@uniroma5.it; 4 Department of Molecular Biology and Genetics, Çanakkale Onsekiz Mart University, Canakkale 17000, Türkiye; sercankarav@comu.edu.tr

**Keywords:** cruciferous vegetables, nutrition, bioactive compounds, glucosinolates

## Abstract

Glucosinolates (GSLs) are sulfur-containing compounds predominantly found in cruciferous vegetables such as broccoli, kale, and Brussels sprouts, and are recognized for their health-promoting properties. Upon consumption, GSLs undergo hydrolysis by the enzyme myrosinase, resulting in bioactive compounds like isothiocyanates and specific indole glucosinolate degradation products, such as indole-3-carbinol (I3C) and 3,3′-diindolylmethane (DIM), which contribute to a range of health benefits, including anti-cancer, anti-inflammatory, and cardioprotective effects. This review explores the structure, metabolism, and bioavailability of GSLs. Recent evidence supports the protective role of GSLs in chronic diseases, with mechanisms including the modulation of oxidative stress, inflammation, and detoxification pathways. Furthermore, the innovative strategies to enhance GSL bioactivity, such as biofortification, genetic introgression, and optimized food processing methods, have been examined. These approaches seek to increase GSL content in edible plants, thereby maximizing their health benefits. This comprehensive review provides insights into dietary recommendations, the impact of food preparation, and recent advances in GSL bioavailability enhancement, highlighting the significant potential of these bioactive compounds in promoting human health and preventing chronic diseases.

## 1. Introduction

Glucosinolates (GSLs) are a prominent group of sulfur-containing compounds predominantly found in cruciferous vegetables such as kale, cabbage, swede, turnip, broccoli, cauliflower, and Brussels sprouts [1]. GSL content varies between cruciferous varieties, plant parts, and growth environments [2]. These bioactive compounds have attracted significant attention due to their potential role in human health, particularly in chronic disease prevention [3]. Upon consumption, GSLs are enzymatically hydrolyzed by myrosinase to produce biologically active compounds such as isothiocyanates and indoles [4], which exhibit a range of health-promoting properties, including anti-cancer, anti-inflammatory, and antioxidant effects [5,6]. Recent research has emphasized the beneficial impact of glucosinolate derivatives, especially sulforaphane, in modulating oxidative stress and inflammation, crucial contributors to the development of chronic conditions such as cardiovascular diseases, diabetes, and cancer. For instance, sulforaphane has been shown to activate the Nrf2 pathway, leading to the increased expression of antioxidant enzymes and reduced inflammatory responses [7]. Epidemiological studies have linked the regular consumption of glucosinolate-rich vegetables with reduced risks of these diseases, highlighting their role in diet-based disease prevention strategies. However, factors such as food preparation and storage methods, along with the metabolic responses of various human organs [8] can significantly influence the bioavailability and efficacy of glucosinolates in the human body. Additionally, glucosinolates contribute to the detoxification of harmful substances in the body by promoting the activity of enzymes involved in liver detoxification processes, thus supporting overall health.

Scientific interest in GSLs has grown significantly, with numerous reviews exploring specific aspects such as their chemical properties, mechanisms of action or effects on individual diseases. However, these reviews often lack an integrated approach linking the chemistry of GSLs, their bioavailability, nutritional improvement strategies, and potential in the prevention of chronic diseases. This review aims to be a comprehensive review that integrates several crucial aspects of GSLs, from their chemical structure and metabolism to their biofortification and effects on human health. The aim is to provide readers with a broad yet articulate overview that combines the latest evidence with an application perspective for nutrition and public health. This overview addresses a practical and scientific need to consolidate key information for researchers, nutritionists, and health professionals who wish to understand the multiple roles of GSLs in a holistic context.

In recent years, several reviews have analyzed the effects of GSL on human health, with a focus on specific aspects. Ishida et al. discussed the metabolism and functionality of GSLs in Brassicaceae, emphasizing the role of genetic selection in optimizing their content in plants [9]. In contrast, Blažević et al. provided an overview of the structural diversity of GSL, describing their chemical synthesis and metabolism processes in plants [10]. Other reviews, such as that by Costa-Pérez et al., focused on the bioavailability and metabolic pathways of GSLs and their bioactive derivatives, summarizing the available clinical evidence [11]. Hanschen et al. examined the factors influencing the formation of isothiocyanates (ITCs) during the enzymatic degradation of GSLs, exploring the optimal conditions to maximize their production [12]. Maina et al. performed a systematic review on the role of GSLs in human health, considering strategies to enhance their bioactivity through nutrition [13]. Finally, Johnson et al. highlighted how GSLs are degraded by myrosinase both in the gastrointestinal tract and in the intestinal flora, with the production of bioactive compounds that can induce phase II enzymes and inhibit tumor cell proliferation [14].

This study is notable for its integrated approach that synergistically analyzes nutrition and the molecular mechanisms underlying the beneficial effects of GSL. Unlike Ishida et al., who focused on agronomic aspects, and Blažević et al., who focused on the synthesis and metabolism of GSL in plants, our work explores the link between the dietary consumption of GSL and human biological responses [10]. Furthermore, compared to Costa-Pérez et al., who mainly dealt with data from clinical interventions, this study delves into the cellular and biochemical mechanisms by which GSLs modulate metabolic and immunological responses [11]. Johnson [14] emphasized the importance of understanding and modulating the chemistry and metabolism of GSLs throughout the food chain, from production to digestion, in order to exploit their anticarcinogenic potential, an aspect that our study further explores with new evidence. Finally, while Hanschen et al. and Maina et al. examined how the bioactivity of LSGs is optimized, our work expands the perspective by evaluating their role in chronic disease prevention and nutritional implications [13]. This approach aims to provide a broader understanding of the potential of LSGs in health promotion and disease prevention, with an innovative and multidisciplinary contribution to the literature.

## 2. Materials and Methods

This narrative review synthesizes the existing literature on GSLs and their derivatives, focusing on health benefits, bioavailability, and the effects of food processing and agricultural practices on the stability of GSLs. This narrative review adheres to strict inclusion criteria to ensure the relevance and robustness of the studies discussed, prioritizing experimental rigor and recent advances. Relevant, peer-reviewed studies were selected through comprehensive searches of databases such as PubMed and Scopus, with search terms including ‘glucosinolates’, ‘isothiocyanates’, ‘cruciferous vegetables’, and ‘chronic disease prevention’. Studies from 1 January 2002 to 1 January 2025 were included. The studies were chosen based on the relevance of metabolism, bioavailability, and health impact of GSLs, as well as those that offered insights into food processing or biofortification methods. To ensure comprehensive coverage, the literature search included studies published in English in various databases. The articles were selected according to specific inclusion criteria: (1) relevance of glucosinolates’ chemical properties, metabolic pathways, and health benefits; (2) experimental rigor, with priority given to studies employing robust methodologies, including in vitro, in vivo, and human studies; and (3) focus on food processing, storage, and agricultural practices that impact the stability of GSLs. Exclusion criteria were focused on studies lacking experimental detail, of minimal relevance to human health, and non-peer-reviewed sources. In case of conflicting results, only studies with the most recent and complete data were included to ensure accuracy and relevance. Data extraction was performed independently by two reviewers to mitigate bias, followed by cross-validation to verify consistency and relevance to the topics explored in this review. The selected literature was organized by the following topics: (1) chemical properties and metabolism, (2) bioavailability and absorption, (3) mechanisms for the prevention of chronic diseases, and (4) impact of food processing and agricultural methods. This structure facilitated an integrated analysis of food. Unlike previous reviews, this work synthesizes findings from different disciplines, integrating biochemical, nutritional, and public health perspectives. This integrative approach bridges the gaps between biochemical properties, bioavailability, and implications for chronic disease prevention, providing a comprehensive perspective for advancing research and practical applications.

## 3. Chemical Properties, Metabolism, Bioavailability, and Absorption

### 3.1. GSLs and Derivatives

GSLs are sulfur-containing, water-soluble glycosides with low molecular weight, synthesized as secondary metabolites in herbaceous plants belonging to the orders Capparales and Brassicales. This diverse group encompasses approximately thirty families. Notably, GSLs are abundant within the Brassicaceae family (commonly referred to as Cruciferae), and to a lesser extent, in the Moringaceae family, particularly in *Moringa oleifera*, the most widely cultivated species [9]. The Brassicaceae family is globally distributed, comprising more than 300 genera and approximately 4000 species. These include numerous species of socio-economic importance and widely consumed vegetables, such as broccoli, cabbage, cauliflower, Brussels sprouts (*Brassica oleracea*), turnips, rapini, Chinese cabbage (*Brassica rapa*), watercress (*Lepidium sativum*), canola (*Brassica napus*), radish (*Raphanus sativus*), arugula (*Eruca sativa*), and capers (*Capparis sicula*). Additionally, several plants within this family are used as condiments, including mustard (*Brassica nigra* and *Sinapis alba*) and wasabi (*Eutrema japonicum*) [15].

GSLs, formerly known as thioglycosides, are water-soluble N-hydroxy sulfates characterized by a sulfur-bound β-D-glucopyranose/β-thioglucose moiety and a sulfonated oxime group. The variation in their structure arises from different side chains derived from amino acids, which form the basis for their classification [8,16]. GSLs are classified into three main groups based on the amino acid-derived side chain: aliphatic group: derived from methionine (Met), alanine (Ala), leucine (Leu), isoleucine (Ile), and valine (Val); indolic group: derived from tryptophan (Trp); and aromatic group: derived from phenylalanine (Phe) and tyrosine (Tyr).

The biosynthesis of GSLs consists of three steps: (i) side-chain elongation of aliphatic amino acids by methyl groups; (ii) formation of a central structure by metabolic reconfiguration of the amino acid moiety; (iii) modification of the central structure by various gluconic structures. The chain elongation step involves the deamination of branched-chain amino acids by aminotransferase, transforming the precursor amino acids into the corresponding 2-oxo acids. Subsequently, the 2-oxo acids are subjected to three enzymatic reactions. First, the acids are condensed with acetyl-CoA by a methylthio alkyl malate synthase, forming a 2-malate derivative, which is isomerized to 3-malate by an isopropylmalate isomerase. Finally, decarboxylation operated by an isopropylmalate dehydrogenase leads to the formation of an elongated 2-oxo acid intermediate. This compound can undergo transamination, providing an elongated amino acid for the next step, or re-enter the transformation cycle for further elongation. The metabolic reconfiguration step begins with the oxidation of the elongated amino acid into the corresponding aldoximes. This oxidation is catalyzed by three enzyme systems: cytochrome-P450-dependent monooxygenase, flavin-containing monooxygenase, and peroxidase. The involvement of the above enzymes depends on the amino acid precursors. The monooxygenases activate the aldoxime to yield the corresponding thiohydroximate. Subsequently, the activated aldoxime is conjugated to glutathione, which acts as a sulfur donor, to produce the thiohydroxime intermediate. The S-alkyl-thiohydroximate intermediate is then cleaved by a C-S lyase to provide the corresponding thiohydroxymates. Finally, these compounds undergo subsequent transformation and desulfoglucosinolate sulfotransferase to obtain the central glucosilonate structure and the corresponding side chains. Transformations of the side chains of GCLs occur through oxidations, eliminations, alkylations, and esterifications catalyzed by enzymes. These modifications contribute to the structural diversity of these molecules, impacting myrosinate-catalyzed hydrolysis and the resulting biological activities of the hydrolysis products [17].

Several studies have shown that the type and quantity of GSLs is not only related to the botanical variety, but can vary between individual plant organs [18] during developmental stages [19] in response to the cultivation and climatic conditions in which the plant lives [20,21]. Studies reported that the distribution is higher in seeds than in leaves in *Brassica napus* [17]. Some studies demonstrate that the amount of GSL in the leaves of curly kale (*Brassica oleracea acephala*) increases during the initial growth stages [22]. Plant adaptation to abiotic stress conditions leads to changes in GSL distribution. For instance, a moderately elevated salinity above tolerance levels results in an increase in these metabolites in broccoli inflorescences. A study conducted on *Brassica rapa* under abiotic stress revealed that the administration of a high salt concentration of NaCl or KCl leads to a decrease in aliphatic GSLs in sprouts, while the administration of Na_2_SO_4_ causes an increase in indolic and aromatic GSLs [23]. Several studies also confirm that water stress can cause either an accumulation or reduction in GSL in some varieties of the Brassica species [24].

The metabolic derivatives of glucosinolates, primarily isothiocyanates, nitriles, and indoles, are bioactive compounds generated from enzymatic hydrolysis in cruciferous plants. When plant tissues are damaged, myrosinase, an enzyme released from cell compartmentalization, acts on glucosinolates, converting them into various biologically active metabolites. The most important and studied class of derived metabolites is that of isothiocyanates (ITCs), substances represented by the general formula R-N=C=S (R-NCS), where R can be an aliphatic, aromatic, or heterocyclic group with different structures. Due to the highly electrophilic nature of the carbon in the -NCS group, ITCs are highly unstable, as well as volatile, and can easily give rise to oxazolidine-2-thiones through spontaneous cyclization. Additionally, the literature reports that in aqueous solution, ITCs can hydrolyze to generate primary amines, which can react with other ITC molecules to form disubstituted thioureas if there is an increase in temperature or pH (e.g., under boiling conditions). Moreover, indolic ITCs are extremely unstable and undergo hydrolysis to form indole-3-carbinol. Under physiological conditions, ITCs can also reversibly react with thiols to form dithiocarbamates [25]. The main examples of ITCs include the following: benzyl isothiocyanate (BITC), a product derived from the glucosinolate glucotropaeolin; phenethyl isothiocyanate (PEITC), derived from gluconasturtiin, both abundant especially in watercress and salad crucifers; allyl isothiocyanate (AITC), derived from sinigrin, which is rich in wasabi; sulforaphane (SFN), whose precursor is glucoraphanin, abundant in broccoli; erucin (ER), derived from glucoerucin, characteristic of arugula; and moringin (MG) from *Moringa oleifera*, whose precursor is glucomoringin [26,27]. Among these, goitrin, derived from the glucosinolate progoitrin, is notable for its goitrogenic properties in animals. However, at the low concentrations found in cruciferous vegetables, it poses no health risks to humans [22]. Indole-3-carbinol (I3C), an indole-based derivative, is another GSL hydrolysis product with significant biological effects. Found in Brassicaceae, I3C is less stable than sulforaphane and primarily metabolizes into 3,3′-diindolylmethane (DIM), which shows promising effects in regulating hormonal activity, particularly in estrogen-dependent tissues. In addition to ITCs, GSL hydrolysis can also yield nitriles, epithionitriles, or thiocyanates, although its effects on human health are not so well studied. Nitriles, for instance, are produced by the action of myrosinase in the presence of epithiospecifier proteins (ESPs), a specific pH, and ion conditions. While nitriles like sulforaphane nitrile and iberin nitrile are detected in human plasma and urine after consuming broccoli sprouts, their benefits are primarily linked to plant defense, with no conclusive evidence of the health effects for humans [24]. Epithionitriles are formed under specific conditions and the key precursors are sinigrin and gluconapine. Thiocyanates, on the other hand, are formed in the presence of thiocyanated proteins, as described by Kuchernig et al. [28], rather than requiring highly basic conditions, which are not commonly found in native plants. Although these compounds are less common in dietary sources, they reflect the diversity of GSL metabolic pathways and the potential influence of plant growth conditions on GSL types and their biological availability [29]. Table 1 provides a comprehensive overview of key metabolites, their precursors, primary food sources, and molecular structures to illustrate the diversity of bioactive metabolites derived from glucosinolates.

This table provides a detailed characterization of bioactive metabolites derived from glucosinolates, listing their specific precursor compounds, molecular structures, and primary dietary sources. Each metabolite is associated with particular cruciferous vegetables, highlighting their potential health benefits and the structural diversity that contributes to their biological activity.

### 3.2. Metabolic Fate of Glucosinolates in Humans: Bioavailability and Absorption

The biological activity of glucosinolates is primarily attributed to their hydrolysis products. When plant tissue is damaged, the enzyme myrosinase catalyzes the breakdown of glucosinolates, releasing glucose and forming various products depending on the structure of the R-group. These include isothiocyanates (ITCs), thiocyanates, and nitriles, with isothiocyanates being the most studied due to their potential anticancer effects [40]. The formation of sulforaphane, an isothiocyanate derived from glucoraphanin, has been particularly linked to cancer prevention and detoxification mechanisms in human cells [41]. Although some cruciferous vegetables are consumed fresh, human consumption of these vegetables predominantly occurs after cooking, leading to the denaturation and inactivation of plant myrosinase. Several studies in the literature address this topic, reporting that enzyme activity is almost completely lost (−97%) after microwaving cabbage for 2 min, whereas steaming for 7 min results in a 90% loss [42]. However, experiments conducted on volunteers who were given cooked broccoli observed that chewing led to the partial hydrolysis of GSLs, demonstrating that, albeit weak, there is still activity by plant myrosinase, as a result of plant tissue damage induced by chewing and direct contact with GSLs [43].

Longer chewing times provided higher levels of ITCs. Some studies have shown that salivary enzymes do not influence GSL metabolism in the mouth and that no enzymatic hydrolysis reactions occur in the stomach. A small portion of GSLs is absorbed intact, while most GSLs reach the intestinal lumen [8]. In the absence of plant myrosinase, denatured due to gastric pH and inactivated by cooking, intact GSLs in the intestine can cross the brush border of enterocytes and enter the bloodstream, possibly via passive diffusion or facilitated transport, or they can be further metabolized through hydrolysis catalyzed by bacterial myrosinases from the gut microbiota [44,45]. Several studies have confirmed the presence of myrosinases in the human microbiota, such as in some Enterococcus, Lactobacillus, Bifidobacterium, and Bacteroides species, which can therefore contribute to hydrolyzing dietary GSLs, albeit to a lesser extent. A study by Liou et al. (2020) has identified myrosinase activity in species of the human microbiota, such as *Bacteroides thetaiotaomicron*, demonstrating their role in hydrolyzing glucosinolates into active metabolites [46].

Another study also showed that *Bacteroides thetaiotaomicron* can metabolize glucosinolates into bioactive isothiocyanates. Gnotobiotic rats associated with a human strain of *Bacteroides thetaiotaomicron* exhibited the formation of allyl isothiocyanate from sinigrin in the digestive tract [47].

This microbial action is significant because isothiocyanates are not formed in the absence of enzymatic breakdown, limiting their health benefits unless gut microbiota is involved [17]. The activity of intestinal bacterial myrosinases has been demonstrated through studies on the bioavailability of glucoraphanin. In these studies, glucoraphanin was administered without plant myrosinase, revealing that its hydrolysis results in a significant loss of glucosinolates, particularly in the small intestine [23]. In another clinical study conducted on patients who were given cooked and raw broccoli and radishes, the detection of sulforaphane in the urine indicated efficient activity by intestinal bacteria. However, further studies are needed to understand the function and activity of intestinal myrosinase on GSL metabolism [22]. The ability of the microbiota to metabolize glucosinolates depends on several factors, including the specific composition of the microbiota. One study showed that subjects with high *Prevotella copri* exhibited increased urinary excretion of isothiocyanates after broccoli consumption, suggesting that specific gut bacteria may modulate the efficiency of glucosinolate metabolism [48].

Individuals with different microbiota compositions, specifically those with higher levels of Bacteroides strains, can exhibit significant differences in isothiocyanate excretion after consuming cruciferous vegetables. Those with higher levels of bacterial strains capable of glucosinolate hydrolysis tend to excrete more isothiocyanates in their urine after consuming cruciferous vegetables [49].

Moreover, the ITCs formed from the hydrolysis of GSLs in the small and large intestines are absorbed by epithelial cells and undergo the conjugation with glutathione (GSH), a phase II detoxification reaction. Subsequently, they enter the bloodstream and are directed to the liver, where they may undergo further transformations through the mercapturic acid pathway, before being excreted in the urine, mainly as N-acetylcysteine-isothiocyanate (ITC-NAC) [8]. The ITC-GSH conjugation occurs at the electrophilic carbon of the isothiocyanate and the nucleophilic thiol group -SH of glutathione and is promoted by glutathione S-transferase (GST). The GSH-conjugated dithiocarbamate undergoes a series of modifications by the sequential action of the enzymes γ-glutamyltranspeptidase (GTP), cysteinylglycinase (CGase), and N-acetyltransferase (NAT) to become mercapturic acids or their derivatives as final products of the metabolic process [47]. One study found that about 60% of ITCs derived from the ingestion of broccoli sprouts are removed via urine within 8 h, and of these, about 65% are in the form of ITC-NAC, 28% as a sulforaphane–cysteine conjugate, 7% as pure sulforaphane, and less than 1% in other conjugated forms [25].

In addition to enzymatic hydrolysis, the gut microbiota metabolizes glucosinolates mainly via redox reactions, favoring the formation of bioactive isothiocyanates, with the results modulated by diet [50].

GSL-derived metabolites are also absorbed in the intestine via passive diffusion or facilitated transport, primarily in the colon, or they are excreted. Similar to what occurs in some herbivores and certain parasites of Brassicaceae, such as *P. xylostella*, which can feed on the plant due to the action of a sulfatase, protecting themselves from the toxicity of isothiocyanates, mammals also use an intestinal sulfatase to rapidly convert ingested GSLs into desulfoglucosinolate [51].

In this metabolic pathway, sulfatase activity seems to favor the formation of nitriles over ITCs [8]. This variability highlights the complexity of glucosinolate metabolism and suggests that diet-induced changes in gut microbiota could modulate the bioavailability of these health-promoting compounds. Further research is necessary to better understand the factors influencing microbial myrosinase activity and its impact on GSL bioavailability, as well as the role of redox reactions and sulfatase in the formation of GSL derivatives. Overall, the interplay between diet, microbiota, and GSL metabolism underscores the importance of individual factors in maximizing the health benefits of cruciferous vegetable consumption

### 3.3. Impact of Food Processing and Cooking Methods on Glucosinolate Stability and Bioavailability in Cruciferous Vegetables

GSLs are relatively inert compounds in plants, but various conditions can lead to chemical or thermal degradation mechanisms similar to enzymatic hydrolysis. Among these are industrial processing and preparation practices, as well as domestic cooking methods, which can significantly affect the stability and thus the bioavailability of these compounds, depending on their intensity and duration. It is essential to control GSL content losses throughout the entire food supply chain, from harvest to consumer delivery, to ensure the highest possible product quality.

Other experiments concluded that cool temperatures between 0 and 4 °C are ideal for preserving product quality. It was also observed that pre-storage at 0 °C for 7–10 days immediately after harvest ensured better preservation of more sensitive crops, such as arugula, which are more susceptible to microbial growth [22]. Another critical factor to control is relative humidity, especially at temperatures above 4 °C, where it is advisable to maintain high levels (98–100%) to preserve cellular integrity [22]. An important aspect in the preservation of glucosinolates is the packaging carried out in the post-harvest period. A study on broccoli (*Brassica oleracea*) showed that the concentration of glucoraphanin decreased by the same percentage, about 55%, in broccoli stored for 3 days in open-air boxes and for 7 days in plastic bags. This concentration was higher in broccoli stored in controlled atmosphere packages than in broccoli stored with air treatment [52]. Additionally, the combination of refrigerated storage with modified atmosphere packaging was more effective than storage in air packs [53].

Furthermore, the composition of the modified atmosphere also affects the components of broccoli heads. A study showed that the total glucosinolate content was better preserved with a 5% CO_2_ + 3% O_2_ atmosphere [54]. Post-harvest freezing has been shown to improve GSL stability and extraction from plant cells, but thawing results in plant tissue breakdown, leading to enzymatic degradation and GSL loss before consumption. However, this drawback can be overcome with a prior quick blanching phase to inactivate myrosinase [55].

However, prolonged heating to 60 °C or higher leads to the denaturation and inactivation of myrosinase [56]. It has been demonstrated that the use of vinegar as a cold seasoning, and to a lesser extent lemon, significantly increases ITC formation, showing that an acidic environment favors GSL degradation, while higher temperatures, such as in boiling, promote the formation of nitriles. Conversely, the addition of baking soda at high temperatures tends to make GSLs more stable [57,58,59]. Experimental studies show that high temperatures significantly degrade GSLs; for instance, boiling broccoli for 10 min reduces glucosinolate content by up to 57% [60]. Their thermolability varies depending on the type of medium that conducts heat (air, water, food matrix, etc.), as well as the type of vegetable used [61]. It has been shown that blanching for five minutes significantly reduced (>50%) GSL content due to cell lysis and leaching. The water from the industrial blanching of cabbage and turnip tops, which is currently discarded in the food industry, has shown a high GSL content and could instead be reused as beverages [22]. Canning, which involves sterilization at temperatures above 100 °C for an extended time (60 min), has been observed to result in GSL losses of up to 80%. Some studies on air drying have revealed that this industrial processing method can lead to a variable reduction in GSLs of between 17% and 45%, depending on the drying temperature (50–100 °C) [56].

The fermentation process used to produce fermented cabbage can change the glucosinolate content. It has been seen that also some lactic acid bacteria perform a similar action to the enzyme myrosinase, degrading glucosinolates. In fact, a study showed that the fermentation of cabbage produces an increase in the content of sub-products such as ascorbigens, isothiocyanates, indole-3-carbinol, and indole-3-acetonitrile, obtained from the degradation of glucobrassicin, sinigrin, and glucoraphanin [62].

In recent decades, the use of various non-thermal technologies in the food industry, such as ultrasound or ultraviolet light treatment, pulsed electric fields, and high-pressure processing (HPP), has enabled not only the necessary microbial inactivation for optimal food preservation but also the maintenance of nutritional quality and organoleptic properties of both fresh and processed products. These technologies have proven to be an effective strategy for cruciferous vegetables, not only preserving the nutritional and health properties of these foods but, in some cases, even improving the content of bioactive compounds beneficial to health [61]. Studies have shown that HPP treatments with a 300 MPa pressure at 20 °C applied to broccoli can preserve a high number of intact GSLs [63]. Specifically, HPP at these conditions preserved over 80% of the glucosinolates in treated broccoli, outperforming conventional methods [64].

Domestic cooking is one of the post-harvest processes that causes the greatest GSL loss from edible vegetables. It has been observed that in fully cooked broccoli, ITC bioavailability is three times lower than in raw or lightly cooked vegetables; moreover, high temperatures favor the formation of nitriles rather than ITCs [65].

Cooking time is also crucial and should be as short as possible to reduce losses and promote ITC formation. A comparative study of different cooking methods found that sulforaphane content in broccoli samples was reduced by 20% after steaming, 36% after frying, and 88% after boiling [66]. Boiling leads to significant cell lysis and loss of glucosinolates, with reductions of up to 60% reported in cabbage and broccoli [32]. Boiling resulted in a loss of up to 57% of GSLs in Brassica vegetables, while steaming preserved these compounds much better [67]. Tiwari et al. [55] developed a model predicting that boiling results in a 50–76% degradation of GSLs, whereas blanching causes only 18–36% losses. This highlights how different cooking methods significantly impact GSL retention. In general, blanching or steaming allows a better preservation of the nutritional qualities of cruciferous vegetables, while boiling, even for short periods (1–10 min) and at temperatures between 50 °C and 100 °C, results in the worst conditions in terms of GSL content due to their water solubility and ease of diffusion into the cooking medium.

Studies on microwave cooking have revealed that this technology can be useful for cooking cruciferous vegetables if used at a low power (540 W) and for short times, in the absence of water, as under these conditions, GSL losses are less than 18% and myrosinase remains active, thus promoting ITC formation over nitriles [68]. Microwave cooking at low power retains 82% of glucosinolates, compared to only 36% when boiling broccoli [66].

A study by Verkerk and Dekker [69] found that intermediate microwave powers (e.g., 540 W for 8 min) resulted in a substantial retention of GSLs and significant myrosinase activity in red cabbage, which supports ITC formation. A recent study applied to five different cruciferous vegetables, including various types of cabbage, Brussels sprouts, and broccoli, revealed that air frying is a good cooking method for these vegetables, particularly for total flavonoid and phenolic content, although GSL losses ranged from 30% to 70% [70]. Another study showed that stir-frying for a few minutes did not alter the glucosinolate content of several vegetables (broccoli, cabbage, cauliflower, and Brussels sprouts). This is because the moderate temperatures and the short times quickly inhibited the activity of myrosinase without altering the glucosinolate content [66].

The available studies show that cooking methods also influence the varying sensitivity of different GSLs depending on the nature of the side chain. A recent study, for example, showed that sinigrin in cabbage samples is more resistant to steaming and pan cooking than glucoiberin [60]. In general, aliphatic GSLs such as those found in broccoli remained relatively unchanged during steaming, but showed a decrease of around 40% when boiled, which is consistent with studies on GSL stability under different cooking conditions [31]. In contrast, indolic GSLs, known for their sensitivity, exhibited significant losses during cooking. For instance, steaming led to 37% losses, while boiling resulted in a 60% reduction, confirming their higher thermolability compared to aliphatic GSLs [71]. However, it has also been demonstrated that the thermal treatment of cruciferous vegetables in the presence of other ingredients in a binary mixture, such as potatoes, corn starch, or onions, reduces GSL degradation, providing benefits not only to the flavor of the dish but also to its healthfulness [61].

## 4. Dietary Sources and Role of Glucosinolates in Chronic Disease Prevention

### 4.1. Dietary Sources and Recommendations for Optimal Intake

Research has shown that GSLs and their breakdown products offer several health-promoting benefits, particularly in reducing the risk of chronic diseases [72]. The Mediterranean diet, rich in cruciferous vegetables, is known for its positive impact on cardiovascular diseases, metabolic disorders, and certain cancers. The bioactive compounds derived from GSLs, such as sulforaphane, exhibit anti-inflammatory, antioxidant, and cancer-preventive properties. Sulforaphane regulates oxidative stress and inflammatory responses by modulating Nrf2 and NF-κB cellular pathways. Moreover, isothiocyanates and indoles are abundant in vegetables such as broccoli, cabbage, Brussels sprouts, and mustard greens. Based on current research, specific dietary recommendations have been established to optimize the health benefits associated with these bioactive compounds [73]. Table 2 illustrates the potential health benefits of glucosinolates and their derivatives, the dietary sources of glucosinolates, the recommended intake for optimal health results and the mechanisms by which these compounds contribute to disease prevention and health promotion. The recommended intake varies between 100 and 300 g per day of cruciferous vegetables, depending on the specific health outcomes to be achieved. An intake of 100–200 g per day of cruciferous vegetables, including broccoli, cabbage, and kale, has been shown to significantly reduce the risk of cancers, particularly lung and gastrointestinal cancers [74]. Similarly, a daily intake of 200 g of broccoli, cauliflower, and Brussels sprouts has been associated with an 8% to 19% reduction in the risk of colorectal and gastric cancers [75].

For cardiovascular health, the consumption of 300 g/day of cruciferous vegetables has shown significant benefits. This level of intake has been shown to reduce systolic blood pressure and improve vascular function in individuals with mildly elevated blood pressure [76]. In addition, smaller amounts, such as 30 g per day of broccoli and radishes, can provide powerful protection against cancers such as colon and breast cancer due to higher concentrations of bioactive compounds like sulforaphane [77].

The role of glucosinolates in reducing inflammation is also supported by current dietary recommendations. An intake of 150–200 g per day of broccoli, mustard, and Brussels sprouts has been shown to reduce markers of inflammation, such as IL-6, CRP, and TNF-α [24]. To achieve these anti-inflammatory effects, the regular consumption of bioactives derived from Brassica, in particular, sulforaphane and allyl isothiocyanate, has been recommended [78].

In conclusion, the supplementation of 100–300 g/day of cruciferous vegetables in the diet, with an emphasis on diversity and including vegetables high in glucosinolates such as broccoli, cabbage, and Brussels sprouts, offers significant health benefits, including cancer prevention, cardiovascular protection, and anti-inflammatory effects. However, the effect seems to come mainly from diet rather than supplements. Recent studies indicate that the bioavailability of sulforaphane and erucin is significantly lower when consuming broccoli supplements compared to fresh sprouts. This finding emphasizes the importance of dietary sources over supplements to maximize the intake of bioactive compounds and their health benefits [79]. Armah et al. (2013) explored how the genotype may influence the response to glucosinolate-rich diets. Their study showed that individuals with certain single nucleotide polymorphisms (SNPs) in the PAPOLG gene exhibited distinct metabolic changes, including alterations in lipid and amino acid metabolites, after consuming HG broccoli [80].

**Table 2:** Health benefits and dietary recommendations of glucosinolates and their bioactive-derivatives from cruciferous vegetables.

Author	Dietary Sources	Derivatives	Recommendations for Optimal Intake	Anti-Cancer Properties	Mechanisms of Anti-Cancer Activity	Anti-Inflammatory Effects	Cardio Protective Effects
Lund E. [74]	Cruciferous vegetables (broccoli, cabbage, kale, Brussels sprouts)	Isothiocyanates, Nitriles	100–200 g/day cruciferous vegetables	↓ cancer risk (lung, gastrointestinal)	Modulates xenobiotic-metabolizing enzymes, induces apoptosis and cell cycle arrest	↓ IL-6, TNF-α	Improves lipid profiles, reduces oxidative stress
Keum Y. [81]	Cruciferous vegetables (broccoli, watercress, Brussels sprouts, cabbage)	Phenethyl-isothiocyanate, Sulforaphane	3–5 servings/week	↓ cancer risk (lung, colorectal)	Inhibits carcinogen activation via CYP450, induces apoptosis via JNK and caspases	Modulates NF-κB and AP-1 pathways	Improves endothelial function, reduces atherosclerosis progression
Murashima M. [82]	Broccoli sprouts	Isothiocyanates	100 g/day	-	-	↓ oxidative stress markers	Improved cholesterol metabolism, ↓ oxidative stress markers
Christiansen B. [83]	Dried broccoli sprouts	Glucosinolates	10 g/day	-	-	=	= endothelial function in hypertensive individuals
Bahadoran Z. [84]	Broccoli sprouts powder	Isothiocyanates	10 g/day	-	-	↓ MDA, oxidized LDL, OSI	Favorable effects on oxidative stress status in Type 2 diabetes patients
Bahadoran Z. [85]	Broccoli sprouts powder	Isothiocyanates	10 g/day	-	-	↓ serum triglycerides, OX-LDL/LDL ratio	Improved lipid profiles, OX-LDL/LDL ratio, and HDL-C in Type 2 diabetes patients
Armah C. [80]	High-glucoraphanin broccoli	Glucoraphanin, Isothiocyanates	400 g HG broccoli/week	↓ cancer risk	Modulates mitochondrial function	Rebalances TCA cycle and FA oxidation	= effect on CVD biomarkers
Armah C. [86]	High-glucoraphanin broccoli	Glucoraphanin, Isothiocyanates	400 g HG broccoli/week	↓ cancer risk	LDL ↓ through glucosinolate derivatives (isothiocyanates)	-	HG broccoli ↓ LDL by 5–7% in RCTs
Sturm C. [78]	Broccoli, cabbage, rocket (arugula)	Sulforaphane, Allyl isothiocyanate (AITC), Erucin	50–100 g/day	↓ cancer risk (colorectal, lung)	Activates Nrf2 pathway, suppresses NF-κB, induces phase II enzymes	↓ IL-6, TNF-α, CRP, COX-2	Improves antioxidant response, reduces inflammatory markers
Johnson I. [75]	Broccoli, cauliflower, kale, Brussels sprouts	Sulforaphane, Indoles, Isothiocyanates	~200 g/day cruciferous vegetables	↓ risk of colorectal and gastric cancers by 8% and 19%, respectively	Modulates phase I and II detoxifying enzymes, induces apoptosis	↓ IL-6, TNF-α, modulation of immune response	Improves vascular health, reduces risk of atherosclerosis
Abellán A. [77]	Broccoli, radish, kale, mustard, pak choi	Sulforaphane, Glucoiberin	30 g/day of sprouts (broccoli/radish)	↓ cancer risk (colon, breast)	Inhibits phase-I enzymes, induces phase-II detoxifying enzymes (Nrf2)	↓ IL-6, TNF-α, CRP by 59%	Modulates oxidative stress, improves lipid profile
López-Chillón M. [87]	Broccoli sprouts	Isothiocyanates	30 g/day	-	-	↓IL-6 and CRP	↓ inflammatory markers in overweight subjects
Sikorska-Zimny K & Beneduce L [23]	Brassica species (*B. nigra*, *B. oleracea*, *B. rapa*, etc.)	Isothiocyanates (e.g., Sulforaphane), Indoles	150–200 g/day of Brassica vegetables	↓ cancer risk (lung, gastrointestinal)	Modulates phase II detoxification enzymes, induces apoptosis	↓ IL-6, CRP, TNF-α	Protects endothelial function, reduces atherosclerosis progression
Langston-Cox A. [88]	Broccoli extract	Sulforaphane	Higher doses needed for efficacy in pregnant women	-	May improve endothelial function and blood pressure	-	Modest ↓ in diastolic blood pressure in women with pregnancy hypertension
Na G. [89]	Broccoli, watercress, kale, mustard	Sulforaphane, Allyl isothiocyanate (AITC)	70 g/day of broccoli sprouts	↓ cancer risk (breast, liver, bladder)	Modulates tumor microenvironment, inhibits glycolysis, regulates self-renewal signaling of cancer stem cells	↓ IL-8, modulates gut microbiota	Protects cardiovascular health via angiogenesis regulation
Connolly E. [76]	Broccoli, kale, cauliflower, cabbage	Glucoraphanin, Sulforaphane	300 g/day of cruciferous vegetables	Reduces risk of cardiovascular events	Activates Nrf2, reduces oxidative stress	↓ markers of inflammation such as F2-isoprostanes	↓ systolic blood pressure compared to root and squash vegetables

Table 2 provides an overview of the health benefits of glucosinolates (GSLs), sulfur-containing compounds found in cruciferous vegetables, and their bioactive derivatives, including allyl isothiocyanate (AITC) and isothiocyanates (ITCs). Acronyms: LDL (low-density lipoprotein), HDL (high-density lipoprotein), IL-6 (interleukin-6), TNF-α (tumor necrosis factor-alpha), CRP (C-reactive protein), NF-κB (nuclear factor kappa-light-chain-enhancer of activated B cells), and Nrf2 (nuclear factor erythroid 2-related factor 2). The table highlights dietary sources, optimal intake recommendations, and the anti-cancer, anti-inflammatory, and cardioprotective effects of glucosinolates and their derivatives.

### 4.2. Role of Glucosinolates in Chronic Disease Prevention

Several studies have examined the impact of glucosinolates, in particular, rapeseed oil, broccoli, and its derivatives, on cardiovascular biomarkers. Rapeseed oil demonstrates significant potential for improving lipid profiles, reducing oxidative stress, and offering anti-inflammatory and endothelial benefits, making it a valuable dietary component for cardiovascular health.

#### 4.2.1. Oxidative Stress and Anti-Inflammatory Effects

Cardiovascular diseases are the leading cause of death today and are caused by multiple factors: physical inactivity, unhealthy diet, smoking, diabetes, hypertension, and obesity [90]. In addition to these medical and lifestyle conditions, oxidative stress plays an important role in the evolution of cardiovascular diseases. ROS and reactive nitrogen species (RNS) are responsible for myocardial lesions that cause impaired cardiac function [91]. Inflammation also contributes significantly to exacerbating the situation, causing death by apoptosis of cardiomyocytes [92]. In recent years, the potential role of some GSLs in the prevention and treatment of cardiovascular diseases has gained attention. Among these, sulforaphane (SFN) has shown the most significant cardioprotective activity [27,93,94,95] due to its ability to modulate oxidative stress, inflammation, and apoptosis. As demonstrated in cancer research, SFN’s control over these signaling mechanisms highlights its potential in preventing and treating CVDs. Here, we will emphasize its role in modulating these signaling pathways and those regulated by adenosine 5′-monophosphate (AMP)-activated protein kinase (AMPK) in protecting against inflammation related to cardiovascular diseases.

In particular, SFN reduced lipid peroxidation and ROS levels in adult cardiomyocytes by 40%, increasing the activity of superoxide dismutase (SOD) mediated by Nrf2 and PGC-1α [96]. It also increased glutathione, the master endogenous antioxidant, by activating the Nrf2/ARE pathway. Therefore, SFN acts as an Nrf2 activator, modifying Keap1 to prevent Nrf2 degradation, thus enhancing the antioxidant defense of cardiomyocytes [97]. The Nrf2 upregulation by the SFN treatment of cardiomyocytes promotes increased expression and activity of Nrf2-associated antioxidant genes, such as NQO-1, HO-1, and GCL, thus contributing to counteracting pathological processes in cardiovascular diseases [98]. Clinical research has underscored the effectiveness of GSLs in reducing oxidative stress markers. Bahadoran et al. (2011) demonstrated that broccoli sprout powder significantly reduced markers of oxidative stress, such as malondialdehyde and oxidized LDL-cholesterol, while increasing total antioxidant capacity in type 2 diabetic patients [84]. Similarly, López-Chillón et al. (2019) found that the long-term consumption of broccoli sprouts in overweight subjects significantly reduced inflammatory markers such as IL-6 and the C-reactive protein [87]. By influencing these pathways, glucosinolates not only reduce the accumulation of ROS but also suppress the expression of pro-inflammatory cytokines such as TNF-α and IL-6. This dual action of antioxidative and anti-inflammatory effects positions glucosinolates as promising agents for the prevention of chronic inflammatory diseases linked to oxidative stress, such as cardiovascular disease [99,100,101]. The topical application of benzyl isothiocyanate (BITC), belonging to the class of GSLs, in psoriasis mice improved 12-O-tetradecanoylphorbol-13-acetate (TPA)-induced swelling in the ears (a model for inflammatory diseases) by decreasing the expressions of iNOS and COX2 [102,103]. Other GSLs (SFN and BITC) are also able to reduce the inflammatory process and the induction of NF-kB, the enhanced intercellular adhesion molecule 1 (ICAM-1), the vascular cell adhesion molecule 1 (VCAM-1), and E-selectin, through the increase in heme oxygenase (HO)-1, glutamate cysteine ligase (GCL) catalytic and modifier subunit expression, and intracellular GSH content [104]. GSLs and their hydrolysis products have excellent anti-inflammatory and antioxidant effects via the Nrf2 activation pathway, a reduction in NF-kB activation, and subsequent reduced cytokine levels. The most studied GSL in the prevention of inflammatory damage is SFN, which is now used in the treatment of inflammatory diseases, such as colitis and rheumatoid arthritis, by suppressing the immune system [105]. Patients with acute lung injury and acute respiratory distress syndrome (ARDS) treated with SFN exhibit improved inflammatory, oxidative status and restored macrophage function [106]. Inflammatory responses are also inhibited by SFN treatment in viral infections such as influenza, hepatitis C, and acquired immunodeficiency syndrome (AIDS), in which the upregulation of Nrf2 and downstream antioxidant enzymes is observed [107].

Furthermore, SFN modulates the MAPK/AP-1/NF-κB pathway, reducing the activation of specific kinases (p38, ERK, JNK) and inhibiting NF-κB, with beneficial effects on inflammation associated with cardiovascular diseases [105]. SFN can reduce, in models of atherosclerosis, the expression of adhesion molecules such as ICAM-1, VCAM-1, E-selectin, and MCP-1 by hindering the binding of monocytes to endothelial cells and reducing ROS as well as the damaging effects of oxidized LDL on the endothelium [104,107,108]. This reduction is connected to the modulation of the Rho A/ROCK/NF-κB pathway in atherosclerotic plaques [109]. In an experimental model of myocardial infarction (MI), SFN has been shown to improve structure and function through the modulation of the MAPK pathway. Specially, SFN treatment is able to significantly lower the levels of pro-inflammatory interleukins (IL-1β, IL-6) and TNF-α, while increasing the activation of ERK1/2, GSK-3β, and PKC in an ischemia–reperfusion model [110]. In addition, SFN increases Nrf2 expression and improves the function of antioxidant enzymes such as NQO-1 and HO-1 in cases of congestive heart failure [111]. SFN, through the suppression of the MAPK pathway and modulation of the expression of GATA4 and 6, improves cardiomyocyte hypertrophy and left ventricular systolic and diastolic functions, thus causing a decrease in parameters including LVEF, LVFS, LVESD, and LVEDD [112]. Finally, it has been demonstrated that treating a rabbit model of heart failure with SFN for 12 weeks results in a decrease in the levels of atrial natriuretic peptide (ANP) and brain natriuretic peptide (BNP), contributing to the attenuation of fibrosis and to the improvement of cardiac function [113]. Erucin (ER) is another GSL with cardioprotective activities. In particular, it has been shown that ER is able to reduce systolic blood pressure levels in hypertensive rats [114]. Furthermore, the ability of ER to inhibit NF-kb activity at the platelet level and also the expression of P-selectin, thromboxane B2, CCL5, transforming growth factor beta (TGF-β), and IL-1β has been highlighted, thus demonstrating its antiplatelet and antithrombotic properties [115,116]. Finally, at the cardiac level, ER is able to increase the content of antioxidant enzymes (SOD, catalase, GSH), thus inhibiting the activation of the transcription factor p53 and preventing functional and histological alterations of cardiomyocytes.

#### 4.2.2. Anti-Cancer Effects of GSLs

In recent years, epidemiological studies have shown that a diet rich in broccoli reduces the risk and progression of various forms of cancer such as the colon, stomach, liver, breast, lung, etc. [26,117,118]. The credit seems to be attributed to isothiocyanates, including sulforaphane [70,119], which appears to have antioxidant, anti-inflammatory, and chemopreventive properties [103,120,121]. The most interesting aspect concerns their ability to inhibit the proliferation of tumor cells by acting directly on proteins involved in tumor initiation and proliferation pathways [122]. In particular, several research groups in recent years have demonstrated how sulforaphane is capable of modulating different metabolic pathways in prostate cancer cells [72]. Núñez-Iglesias et al. (2019) highlight a reduced viability of prostate cancer cells (40–60%) when treated for 72 h with 30 μM of sulforaphane. In addition, this treatment also decreased the ability of cancer cells to metastasize by up to 50% [120]. Singh et al. (2018) show in prostate cancer cell lines and in mouse models how treatment with sulforaphane significantly reduces the expression of hexokinase II and pyruvate kinase M2, glycolytic enzymes involved in tumor progression [121]. Sulforaphane also appears to play an essential role in inducing apoptosis in prostate cancer cells according to Ahmed et al. (2018), who reveal that sulforaphane treatment blocks the activity of two deubiquitinating enzymes, USP14 and UCHL5, thus causing an accumulation of polyubiquitinated proteins and ultimately apoptosis [122]. Another study by Rutz et al. (2020) shows that sulforaphane also leads to a reduced proliferation of prostate cancer cells, inducing a block of their cell cycle in the S and G2/M phases [123]. Singh and colleagues (2018) also performed in vivo studies using Transgenic Adenocarcinoma of Mouse Prostate (TRAMP) mice treated with 6 μmol/mouse of sulforaphane. This treatment led to a decrease in prostate tumor formation, along with a 60–70% decrease in the enzymes acetyl-CoA carboxylase (ACC) and fatty acid synthase (FASN) [124]. These enzymes are involved in the synthesis of fatty acids, which are abundantly synthesized during prostate tumor growth.

SFN treatment has been shown to demethylate the nuclear factor-erythroid factor 2-related factor-2 (Nrf2) gene promoter, leading to increased expression in prostate cancer cells [125], which is reflected in decreased tumor cell proliferation [126]. Under normal conditions, Nrf2 is retained by the protein Keap1 (Kelch-like ECH-associated protein), which keeps it ubiquitinated and promotes its degradation. However, in the presence of oxidative or electrophilic stress, Nrf2 separates from the Nrf2-Keap1 complex and rapidly translocates to the nucleus where it binds to the (ARE) region of genes involved in antioxidant and detoxification enzyme production. This process is critical for glutathione (GSH) synthesis, ROS regulation, and drug metabolism [127]. After demethylation, Nrf2 can translocate to the nucleus and promote the transcription of genes, such as NQO1 (NAD(P)H quinone dehydrogenase 1), HO-1 (heme oxygenase-1), and GCLC (glutamate-cysteine γ-ligase) [128].

Several studies have also been conducted in humans to confirm the effects of glucosinolates. Traka et al. (2019) administered broccoli soup once a week to a cohort of prostate cancer patients (subjected to an active surveillance monitoring system). The authors observed a decrease in molecular pathways and gene expression associated with the development and progression of prostate cancer [129]. Similarly, Zhang et al. (2019) showed a decrease in the expression of two prostate cancer-associated genes, AMACR and ARLNC1, by consuming 200 μmol of broccoli sprout extract in a cohort of patients undergoing prostate biopsy [130]. Recently, Zhang et al. (2024) demonstrated that sulforaphane treatment of prostate cancer cells causes an activation of miRNA-3919, which ultimately targets DJ-1, an oncogenic gene. The increased expression of miRNA-3919 not only causes the downregulation of DJ-1 mRNA and protein, but also prevents the migration, viability, and invasion of prostate cancer cells [131].

Treatment with isothiocyanates, particularly sulforaphane, is also associated with breast cancer regression. Major studies highlight its ability to block the cycle of breast cancer cells in the G2/M phase, leading to their death by apoptosis [132]. This block in the G2/M phase would also cause an alteration in the cytoskeleton and tubulin polymerization, resulting in a mitotic catastrophe of tumor cells [133,134]. Furthermore, sulforaphane is able to modify cysteine residues of the promyelocytic leukemia protein (which promotes cell proliferation of MCF-7 breast cancer cells), causing cytotoxicity [135]. Additionally, several authors have emphasized that sulforaphane treatment modifies the activation of the intrinsic apoptotic signaling cascade. Different concentrations of sulforaphane seem to reduce the levels of the Bcl-2 protein and increase those of Bax, inducing the apoptotic death of MCF-7, Bt-474, and T47D cells [136,137,138]. Furthermore, Sarkar et al. found not only a decrease in Bcl-2 and a concomitant increase in Bax and Bad expression in p53 wild type MCF-7 cells after sulforaphane treatment but also the induction of apoptosis mediated by heat shock proteins HSP27, HSP70, HSP90, and HSF1 (heat shock factor 1), with the subsequent upregulation of p21 [139]. In addition to the apoptotic process, some studies have also examined the ability of sulforaphane to modulate the autophagic process in breast cancer cells. Indeed, Kanematsu et al. (2010) observed an increase in the number of autophagosomes, autolysosomes, and LC3-II proteins in MDA-MB-231 cells following sulforaphane treatment for 24 or 72 h [140].

The process of metastasis also seems to be controlled by sulforaphane. Specifically, the treatment alters cell migration and the expression of epithelial–mesenchymal transition markers, which play a crucial role in the metastatic spread of breast cancer [141]. Atwell et al. (2015) investigated the effect of a supplement containing a standardized amount of glucoraphanin along with the enzyme myrosinase (BroccoMax) versus a placebo, administered for at least two weeks to women with abnormal mammograms scheduled for breast biopsies. The results showed that BroccoMax supplementation significantly reduced tissue biomarkers, such as Ki-67 and HDAC3, in benign tissue, but not in ductal carcinoma in situ (DCIS) or invasive ductal carcinoma tissue [142]. Zhang et al. (2016) analyzed the relationship between cruciferous vegetable consumption and biomarkers in women undergoing breast biopsies, finding a negative association between cruciferous vegetable consumption and cell proliferation in DCIS tissue [143], suggesting a potential role for sulforaphane in breast cancer prevention.

Sulforaphane plays an important role in the treatment of hepatocellular carcinoma [144]. In fact, Liu et al. (2017), treating HepG2 hepatocellular carcinoma cells with sulforaphane, observed a decrease in the migration and adhesion of cancer cells as well as a block in the expression of molecules that induce angiogenesis, such as VEGF, STAT3, and HIF-1α [145]. Sulforaphane was also able to induce apoptosis in HepG2 cells by downregulating Bcl-2 and Bcl-XL [140]. More recently, Zou et al. (2017) showed that endoplasmic reticulum (ER) stress is involved in sulforaphane-induced apoptosis. In detail, C/EBP and XBP-1 (ER stress biomarkers) were upregulated in sulforaphane-treated HepG2 cells undergoing apoptosis [146]. In Hep3B cells, sulforaphane reduced cell survival and interfered with telomerase activity by decreasing telomerase reverse transcriptase expression through an ROS-related mechanism. This indicates a novel pathway for the antitumor action of SFN [147].

Unfortunately, in vivo studies on HCC (hepatocellular carcinoma) and sulforaphane treatment are very limited. Athymic Balb/c nude mice xenografted with HepG2 cells and treated with sulforaphane for 13 days showed a decrease in tumor growth and volume [148]. Another in vivo study instead evaluated the effect on mice after providing a diet supplemented or not with broccoli. The supplemented diet significantly blocked the growth of liver tumors [149].

The antitumor effect of glucosinolates has also been examined in relation to colon cancer, in which a diet rich in fiber and vegetables plays an undisputed role. Indeed, sulforaphane treatment in HCT-116 human colon cancer cells induces DNA damage and subsequent death by apoptosis with the release of cytochrome C [150]. Liu et al. (2016) also demonstrated that sulforaphane causes ROS production and cell cycle arrest in HCT 116 human colon cancer cells in the G2/M phase [151]. Sulforaphane also reduced the main markers of colorectal cancer stem cells, such as CD44 and CD133, in HCT116 and SW480 spheroids, through the involvement of the TAp63α/Lgr5/β-catenin pathway [152]. This indicates that exposure to sulforaphane could decrease the proliferative activity of cells.

Even in the case of colon cancer, there are few studies performed with sulforaphane in vivo. However, it has been observed that in patients and mice with colon cancer, sulforaphane treatment causes a reduction in the development of metastases, suggesting that broccoli consumption could block the progression of this type of cancer [91]. Other studies report a significant reduction in aberrant cryptic foci in mice with colon tumors after treatment for up to 24 weeks with sulforaphane [153].

## 5. Strategies for Increasing Glucosinolate Content in Plants

### 5.1. Genetic and Molecular Approaches

Genetic and molecular approaches have shown significant promise in manipulating glucosinolate (GSL) biosynthesis and distribution within plants. Studies on glucosinolate transporters, such as GTR1 and GTR2, have revealed their essential role in the long-distance transport of glucosinolates [154]. Mutations in these transporters, particularly the double mutant gtr1 gtr2, led to an overaccumulation of glucosinolates in leaves and silique walls, highlighting their critical role in tissue-specific distribution. The transporters UMAMIT29, UMAMIT30, and UMAMIT31 were identified as key exporters that facilitate the loading of glucosinolates into the phloem for seed accumulation. Combined mutations in these transporters significantly reduced glucosinolate levels in seeds, suggesting that the precise genetic manipulation of the GTR and UMAMIT transporters could be a powerful strategy to improve the nutritional profile of brassica crops without altering plant-level distribution [155].

Other approaches include genetic introgression, a selection method involving the transfer of genes from one population to another through backcrossing. This technique has been used to improve the bioactivity of glucosinolates by targeting specific alleles and transcription factors related to their biosynthesis. For example, the introgression of a new MYB28 allele from *Brassica villosa* into broccoli cultivars successfully increased glucoraphanin, a bioactive glucosinolate with anti-cancer properties [156].

**Figure 1:**
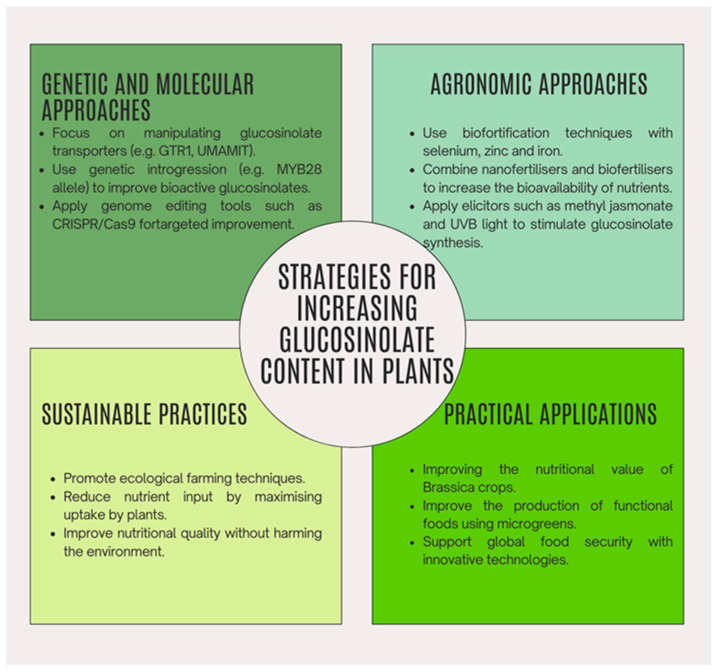
Overview of strategies to increase glucosinolate content in plants, highlighting genetic and molecular approaches (e.g., transporter manipulation, genetic introgression, genome editing) and agronomic and technical approaches (e.g., biofortification, nanofertilizers, elicitation techniques). These strategies aim to improve both the nutritional value and functional properties of Brassica crops.

Figure 1 provides a visual summary of the main strategies for increasing glucosinolate content in plants, divided into genetic/molecular and agronomic/technical approaches.

### 5.2. Agronomic and Technical Approaches

Agronomic and technical approaches encompass a wide range of strategies to improve the glucosinolate content of crops through optimized agricultural practices and innovative technologies. These methods focus on external interventions, such as nutrient supplementation, soil management, and advanced fertilization techniques, to enhance both the nutritional value and functional properties of plants. In particular, biofortification and the integration of nanotechnology have emerged as sustainable and effective solutions to achieve these goals.

Biofortification is an advanced agricultural strategy aimed at enhancing the nutritional profile of crops, particularly through nanofertilizers and biofertilizers, which have demonstrated substantial effects on both plant growth and the accumulation of bioactive compounds such as glucosinolates. The most commonly used mineral in biofortification practices related to GSLs is selenium, a crucial trace element for humans and other organisms, with which the global population is deficient [157]. Selenium biofortification increases the concentration of this mineral in the plant, influencing the production of selenoglucosinolates. The application of selenium fertilizers to the soil also enhances the bioavailability of the mineral when supplied in organic forms, such as selenium amino acids like selenocysteine (SeCys) and selenomethionine (SeMet), compared to inorganic fertilizers (selenates SeO_4_^2−^ and selenites SeO_3_^2−^). Studies have shown that the fertigation of greenhouse-grown broccoli plants with an organic mixture of SeCys and SeMet resulted in a significant increase in the GSLs of plants compared to inorganic forms [158]. Schiavon et al. (2016) investigated the impact of selenium biofortification on radish plants, focusing on its effects on glucoraphanin. They found that selenium application, particularly in foliar sprays and hydroponic systems, significantly enhanced the production of selenium-methylselenocysteine (SeMSCys) and increased the total GSL content by 35%. This study emphasizes the dual benefit of selenium biofortification in enhancing both GSLs and selenium content, making radish a nutritionally superior crop [159]. Moreover, a significant study conducted by Guardiola-Márquez et al. (2023) investigated the effects of combining zinc oxide (ZnO) and iron oxide (γ-Fe_2_O_3_) nanofertilizers with biofertilizers on the growth and glucosinolate content of broccoli microgreens [160]. In this study, zinc and iron nanoparticles, each synthesized with average diameters of 77 nm and 68 nm, respectively, were applied to plants in conjunction with bacterial consortia designed to promote plant growth. The results showed a significant enhancement in micronutrient content, the zinc content rose by 122.19–363.41%, whereas iron content increased by 55.19–161.57% [160]. Most notably, the study observed significant increases in glucosinolate levels. Among the eight glucosinolates detected, those most positively affected by the treatments included glucoraphanin and glucoerucin, which are known for their cancer-preventive properties. The glucosinolate content was found to increase significantly across treated plants, demonstrating that the synergistic interaction between biofertilizers and nanofertilizers can effectively enhance the bioactivity of these compounds.

Nanofertilizers and biofertilizers represent innovative approaches in sustainable agriculture, offering significant advantages in improving crop growth, nutrient content, and the accumulation of bioactive compounds, particularly glucosinolates. Barrameda-Medina et al. (2017) explored zinc biofortification in *Brassica oleracea* cv. Bronco and demonstrated that zinc supplementation (80–100 µM Zn) increased GSL levels along with other phytochemicals. The study revealed that optimal zinc concentrations not only enhanced the biofortification of edible parts but also boosted enzymatic activity related to sulfur assimilation, crucial for GSL biosynthesis. This reinforces the potential of zinc as a critical micronutrient in GSL biofortification programs [161]. In addition to these growth and nutritional improvements, the study demonstrated that biofortification with ZnO and γ-Fe_2_O_3_ nanofertilizers combined with biofertilizers provides a sustainable alternative to traditional fertilization methods by reducing the overall input of nutrients while maximizing their bioavailability and uptake by plants. These findings highlight the potential of nanofertilization to significantly improve both the growth and nutritional quality of crops, particularly in controlled environments where microgreens can be used as functional foods enriched with glucosinolates. The incorporation of nanotechnology into agricultural practices thus represents a critical step toward meeting global food security demands while simultaneously enhancing the health benefits of commonly consumed vegetables.

### 5.3. Genetic Introgression

Genetic introgression, a breeding technique involving the transfer of genes from one population to another through repeated backcrossing, has been increasingly utilized in plant improvement strategies. One of its promising applications is enhancing the bioactivity of glucosinolates. Several recent studies have demonstrated the effectiveness of genetic introgression in modifying the glucosinolate content in crops, particularly in *Brassica juncea* and *Brassica oleracea*, by targeting specific alleles and transcription factors linked to glucosinolate biosynthesis. One of the significant advancements in this field is the use of quantitative trait loci (QTL) mapping to identify genetic loci controlling glucosinolate content. In *Brassica juncea*, the introgression of low-glucosinolate alleles from the East European gene pool into Indian varieties through recurrent selection backcrossing demonstrated the influence of epistatic interactions. This method enabled the identification of “true” QTLs associated with glucosinolate biosynthesis, which are crucial for marker-assisted breeding [162]. Moreover, broccoli cultivars with increased levels of GSLs derived from methionine have been generated through the introgression of a new allele, originating from the *Brassica villosa* species, of MYB28, a transcription factor of aliphatic GSL biosynthesis [163]. Studies have revealed that this introgression results in the upregulation of genes involved in primary sulfate assimilation and sulfur metabolism, leading to the increased accumulation of glucoraphanin, a bioactive glucosinolate with strong anticancer properties [163]. Additionally, research has demonstrated that applying exogenous phytohormones like methyl jasmonate and salicylic acid can further boost glucosinolate levels in Brassica subspecies such as cabbage, broccoli, and kale. This treatment induces the expression of key biosynthetic genes, leading to a significant increase in both aliphatic and indolic glucosinolates, depending on the species [164]. However, the applications of these molecular engineering practices remain limited, primarily due to current market restrictions on genetically modified crops [165]. Concerns have been raised regarding transgenic crops, particularly about the potential risks to human health in terms of toxicity and allergenicity, and to the environment due to biodiversity loss. Additionally, the presence of transgenic crops can cause, through natural pollination, the escape of transgenes to unwanted plants, which through hybridization could create invasive crops with no agricultural advantage and damage useful crops for production [166]. Moreover, there is concern that transgenic foods containing antibiotic-resistant genes could contribute to the phenomenon of antibiotic resistance [167]. Therefore, further improvements and optimizations are necessary for the use of such techniques to maximize the GSLs content in the desired plant organs or tissues, while minimizing any undesirable or harmful effects [165].

A further approach to increase the glucosinolate content in plants is to mutate specific transporters, as demonstrated in *Arabidopsis* and *Brassica napus*. Recent studies have identified the transporters GTR1 and GTR2 as essential for the long-distance transport of glucosinolates to the seeds. In particular, the gtr1 gtr2 double mutant showed an over-accumulation of glucosinolates in the leaves and walls of siliquas, indicating their crucial role in regulating tissue distribution [168]. Similarly, the transporters UMAMIT29, UMAMIT30, and UMAMIT31 have recently been identified as key exporters in seeds, contributing to glucosinolate loading in the phloem [168]. The combined mutation of these transporters significantly reduced the level of glucosinolates in seeds, without drastically altering the overall distribution of the compounds in the plant [155]. These results suggest that the genetic manipulation of the GTR and UMAMIT transporters could be a promising strategy to improve the nutritional profile of brassicaceous crop seeds.

### 5.4. Metabolic Engineering

In recent years, metabolic engineering has been increasingly applied to enhance the bioactivity of GSLs, compounds found in Brassica crops. Glucoraphanin, a major bioactive glucosinolate, has been the target of metabolic engineering in *Brassica oleracea* species. One study utilized coronatine, an elicitor that activates key genes in the glucosinolate biosynthesis pathway, including Cyp79b2, Cyp83b1, Myb51, and Myb122. Specifically, in broccoli cell cultures treated with coronatine, glucosinolate production increased by 205-fold compared to untreated cells, showing a direct correlation between gene expression and enhanced glucosinolate levels [169]. Another strategy involved suppressing the AOP2 gene in *Brassica oleracea* var. alboglabra (Chinese kale) through antisense RNA technology. AOP2 catalyzes the conversion of glucoraphanin into gluconapin, a less desirable glucosinolate. The inhibition of AOP2 significantly increased glucoraphanin levels in transgenic Chinese kale without affecting other key nutrients like vitamin C and carotenoids, indicating the effectiveness of metabolic engineering in improving glucosinolate profiles [170]. A broader study reviewed metabolic engineering approaches in Brassica crops aimed at enhancing beneficial GSLs while reducing anti-nutritional ones. The techniques discussed include altering biosynthesis pathways, inhibiting glucosinolate hydrolysis, and redirecting metabolic flux toward desired compounds. Advances in omics technologies, including genomics and metabolomics, have provided valuable insights into the complex regulation of glucosinolate metabolism [171].

### 5.5. Use of Microgreens and Pre-Harvest Light Application

Among the Brassicaceae microgreens are broccoli (*Brassica oleracea* var. italica), red cabbage (*B. oleracea* var. capitata), daikon or pink Chinese radish (*Raphanus raphanistrum* subsp. sativus), arugula (*Eruca sativa*), mustard (*Brassica juncea*), and watercress (*Nasturtium officinale*) [172]. Microgreens can be cultivated on soil or without soil in urban or peri-urban settings, simply, quickly, and economically, even indoor (vertical) agriculture with hydroponic, aquaponic, aeroponic, or soil-based systems. An additional advantage is that, due to the short production time, these seedlings are generally not susceptible to pest attacks, allowing for pesticide-free cultivation, which is an increasing concern in modern agriculture. These vegetables can also be produced by consumers at home, offering an interesting and sustainable alternative, also reducing food waste due to the sale through large distribution supply chains [173]. In addition to the health benefits, microgreens are a promising eco-sustainable approach to functional foods, having favorable cultivation conditions and a low environmental impact, and they can increase the content of beneficial phytochemicals by up to 10 times compared to mature plants [120]. It is expected that their global sales will grow at an annual rate of over 10% from 2021 to 2028. It has been shown that the pre-treatment of seeds and cultivation at a certain density optimizes growth, leading to better yields in nutritional terms [174]. Enhancing the levels and bioactivity of GSLs in microgreens through pre-harvest light applications and other cultivation techniques is a growing area of research. Calcium chloride (CaCl_2_) application before harvest has been shown to increase glucosinolate concentrations significantly in broccoli microgreens. A metabolomic study demonstrated that treatment with 10 mM CaCl_2_ not only improved shelf-life but also boosted both aliphatic and indolic glucosinolates. This treatment led to enhanced levels of compounds such as glucoraphanin and glucobrassicin, known for their health benefits [174].

Moreover, UVB irradiation before harvest has also been identified as an effective method to increase glucosinolate content. Research demonstrated that UVB treatments at various intensities significantly increased total glucosinolate levels, particularly glucoraphanin and glucoerucin. Furthermore, UVB treatment helped maintain the nutritional quality of broccoli microgreens during post-harvest storage, reducing the degradation of GSLs. Specifically, the nutritional quality of these UV-B-treated vegetables remained stable for a longer time. Additionally, it was observed that adding calcium chloride spray, along with UV-B application, further intensifies the GSLs’ synthesis process, especially aliphatic and indolic GSLs, by almost 70% in treated microgreens, improving their storability and extending post-harvest quality [175]. However, some experiments conducted on Chinese kale (*B. oleracea* var. alboglabra) have shown that the application of UV-A light before harvest increases the total GSL amount and that of aliphatic GSLs, but decreases the presence of some aromatic GSLs and progoitrin, thus having negative effects on their biosynthesis [176]. Nevertheless, the existing discrepancies between the results obtained on the specific amounts of GSLs biosynthesized at different wavelengths indicate the need for further investigation to understand the mechanisms involved in modulating the metabolic processes of individual GSLs through LED lighting and the light intensities to apply [177].

## 6. Strengths, Limitations, and Future Directions

Compared to recent reviews by Zhao et al. [178], Zhang et al. [179], and Sharma et al. [180], this work uniquely integrates the chemical diversity of glucosinolates with biofortification strategies and implications for human health, providing a broader perspective not previously addressed. Zhao et al. (2024) focused on the efficacy of glucosinolates and their metabolites in inflammatory bowel disease, mainly exploring their role in microbiota modulation and antioxidant activity [178]. However, our study extends this approach to include a detailed analysis of the epigenetic pathways and modulatory potential of indole compounds, not covered in that review. Zhang et al. (2024), on the other hand, provided an overview of sulforaphane, highlighting its biosynthesis, metabolism, and clinical applications, while our work focuses on a broader range of compounds derived from glucosinolates, emphasizing their chemical diversity and related health benefits [179].

Sharma et al. (2024) discussed the role of glucosinolates in plant defense and animal health, emphasizing the risks associated with goitrogenic compounds. In contrast, focusing specifically on human health benefits, this review fills gaps in the literature by addressing bioavailability issues, stability during food processing, and innovative approaches such as light treatments and biofortification [180]. These differences highlight the unique contribution of our work, which complements and extends the existing literature. This review highlights the translational potential of glucosinolates and their derivatives in the prevention of chronic diseases, offering insights for dietary strategies and public health recommendations. However, several limitations in current research require attention. A key limitation is the variability in the bioavailability of GSLs between individuals, influenced by factors such as genetic differences, gut microbiota composition, and food preparation methods. Although numerous studies explore the metabolic pathways of GSLs, more human-focused research is needed to clarify how these factors influence the absorption and efficacy of GSLs. Furthermore, while in vitro and animal studies provide valuable mechanistic insights, translating these findings into human health outcomes remains a challenge, particularly for chronic disease prevention. Future research should prioritize clinical studies to better understand the interaction between genetic factors, gut microbiota, and dietary glucosinolate intake. Additionally, exploring sustainable agricultural and food practices could improve the efficacy and accessibility of glucosinolate-rich foods. Finally, investigating the role of innovative pre-harvest and post-harvest technologies, such as light treatments and non-thermal processing, could pave the way for maximizing the health potential of crucifers in a sustainable way.

## 7. Conclusions

Glucosinolates and their derivatives offer a promising natural strategy for the prevention of chronic diseases. This review emphasizes the importance of optimizing their bioavailability and efficacy through innovative agricultural and food processing practices. Future research should focus on customized approaches, considering genetic and microbiota variability, as well as the advancement of sustainable methods to improve the accessibility and health benefits of glucosinolate-rich vegetables. Incorporating these compounds into dietary recommendations is a practical and impactful means to improve public health.

## Figures and Tables

**Table 1:** Characterization of bioactive glucosinolate-derived metabolites: precursors, dietary sources, and molecular structures.

Glucosinolate Metabolite	Precursor Glucosinolate	Chemical Structure ^1^	Primary Source
Sulforaphane (SFN)	Glucoraphanin	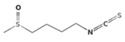	Broccoli, broccoli sprouts [30,31]
Allyl isothiocyanate (AITC)	Sinigrin	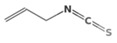	Mustard, wasabi [32]
Benzyl isothiocyanate (BITC)	Glucotropaeolin	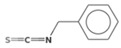	Papaya seeds, mustard [33]
Phenethyl-isothiocyanate (PEITC)	Gluconasturtiin	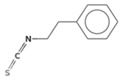	Watercress [24]
Erucin (ER)	Glucoerucin	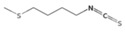	Arugula, broccoli [30]
3,3′-Diindolylmethane (DIM)	Indole-3-carbinol	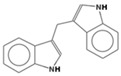	Cabbage, broccoli [30]
Goitrin	Progoitrin	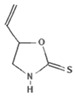	Mustard greens, turnips [34]
Sulforaphane nitrile	Glucoraphanin	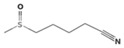	Broccoli, cabbage [30,35]
Iberin nitrile	Glucoiberin	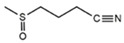	Broccoli, watercress [36,37]
Raphasatin	Glucoraphasatin	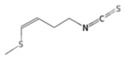	Radish, daikon [37]
Epithionitrile compounds	Sinigrin, Gluconapin	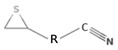 R: alkyl group	Various crucifers [38]
Thiocyanates	Various GSL (e.g., benzyl, allyl GSL)	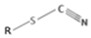	Rare in dietary plants [38]
Moringin (4-(α-L-rhamnopyranosyloxy)-benzyl ITC)	Glucomoringin	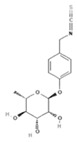	Moringa seeds, leaves [39]

^1^ Chemical structures were obtained from the online database of NIST (National Institute of Standards and Technology) and NIH (National Library of Medicine).

## Data Availability

No new data were created or analyzed in this study. Data sharing is not applicable to this article.

## Abbreviations

The following abbreviations are used in this manuscript:

AITC	Allyl Isothiocyanate
AMPK	Adenosine 5′-Monophosphate-Activated Protein Kinase
ARE	Antioxidant-Responsive Element
CR	Controlled Release
CRP	C-Reactive Protein
FA	Fatty Acid
GCLC	Glutamate-Cysteine γ-Ligase
GSH	Glutathione
GSLs	Glucosinolates
HCC	hepato cellular carcinoma
HDAC	Histone Deacetylase
HDL	High-Density Lipoprotein
HO-1	Heme Oxygenase-1
HSP	Heat Shock Protein
IL-6	Interleukin-6
ITC	Isothiocyanate
LDL	Low-Density Lipoprotein
NF-κB	Nuclear Factor kappa-light-chain-enhancer of activated B cells
NQO1	NAD(P)H Quinone Dehydrogenase 1
Nrf2	Nuclear Factor Erythroid 2–related factor 2
OS	Oxidative Stress
PGC-1α	Peroxisome Proliferator-Activated Receptor Gamma Coactivator 1-alpha
ROS	Reactive Oxygen Species
RNS	Reactive Nitric Oxide Species
SFN	Sulforaphane
SOD	Superoxide Dismutase
TCA	Tricarboxylic Acid (Cycle)
TNF-α	Tumor Necrosis Factor-Alpha

## References

[1] Akram M., Jabeen F., Riaz M., Khan F.S., Okushanova E., Imran M., Shariati M.A., Riaz T., Egbuna C., Ezeofor N.J. (2021). Health Benefits of Glucosinolate Isolated from Cruciferous and Other Vegetables. Preparation of Phytopharmaceuticals for the Management of Disorders.

[2] Ciska E., Martyniak-Przybyszewska B., Kozlowska H. (2000). Content of glucosinolates in cruciferous vegetables grown at the same site for two years under different climatic conditions. J. Agric. Food Chem..

[3] Teodoro A.J. (2019). Bioactive Compounds of Food: Their Role in the Prevention and Treatment of Diseases. Oxidative Med. Cell. Longev..

[4] Becker T., Juvik J. (2016). The Role of Glucosinolate Hydrolysis Products from Brassica Vegetable Consumption in Inducing Antioxidant Activity and Reducing Cancer Incidence. Diseases.

[5] Olayanju J.B., Bozic D., Naidoo U., Sadik O.A. (2024). A Comparative Review of Key Isothiocyanates and Their Health Benefits. Nutrients.

[6] Prado N.J., Ramirez D., Mazzei L., Parra M., Casarotto M., Calvo J.P., Cuello Carrión D., Ponce Zumino A.Z., Diez E.R., Camargo A. (2022). Anti-Inflammatory, Antioxidant, Antihypertensive, and Antiarrhythmic Effect of Indole-3-Carbinol, a Phytochemical Derived from Cruciferous Vegetables. Heliyon.

[7] Chen M., Huang L., Lv Y., Li L., Dong Q. (2021). Sulforaphane protects against oxidative stress induced apoptosis via activating SIRT1 in mouse osteoarthritis. Mol. Med. Rep..

[8] Shakour Z., Shehab N., Gomaa A., Wessjohann L., Farag M. (2022). Metabolic and Biotransformation Effects on Dietary Glucosinolates, Their Bioavailability, Catabolism and Biological Effects in Different Organisms. Biotechnol. Adv..

[9] Ishida M., Hara M., Fukino N., Kakizaki T., Morimitsu Y. (2014). Glucosinolate metabolism, functionality and breeding for the improvement of Brassicaceae vegetables. Breed. Sci..

[10] Blažević I., Montaut S., Burčul F., Olsen C.E., Burow M., Rollin P., Agerbirk N. (2020). Glucosinolate structural diversity, identification, chemical synthesis and metabolism in plants. Phytochemistry.

[11] Costa-Pérez A., Núñez-Gómez V., Baenas N., Di Pede G., Achour M., Manach C., Mena P., Del Rio D., García-Viguera C., Moreno D. (2023). Systematic Review on the Metabolic Interest of Glucosinolates and Their Bioactive Derivatives for Human Health. Nutrients.

[12] Hanschen F.S., Klopsch R., Oliviero T., Schreiner M., Verkerk R., Dekker M. (2017). Optimizing isothiocyanate formation during enzymatic glucosinolate breakdown by adjusting pH value, temperature and dilution in Brassica vegetables and Arabidopsis thaliana. Sci. Rep..

[13] Maina S., Misinzo G., Bakari G., Kim H.Y. (2020). Human, animal and plant health benefits of glucosinolates and strategies for enhanced bioactivity: A Systematic Review. Molecules.

[14] Johnson I.T. (2002). Glucosinolates in the human diet. Bioavailability and implications for health. Phytochem. Rev..

[15] Ağagündüz D., Şahin T.Ö., Yılmaz B., Ekenci K.D., Özer Ş.D., Capasso R. (2022). Cruciferous Vegetables and Their Bioactive Metabolites: From Prevention to Novel Therapies of Colorectal Cancer. Evid. Based Complement. Altern. Med..

[16] Bellostas N., Sorensen J., Sorensen H. (2007). Profiling Glucosinolates in Vegetative and Reproductive Tissues of Four Brassica Species of the U-Triangle for Their Biofumigation Potential. J. Sci. Food Agric..

[17] Nguyen V., Stewart J., Lopez M., Ioannou I., Allais F. (2020). Glucosinolates: Natural Occurrence, Biosynthesis, Accessibility, Isolation, Structures, and Biological Activities. Molecules.

[18] Fahey J.W., Zalcmann A.T., Talalay P. (2001). The Chemical Diversity and Distribution of Glucosinolates and Isothiocyanates among Plants. Phytochemistry.

[19] Cartea M.E., Velasco P. (2008). Glucosinolates in Brassica Foods: Bioavailability in Food and Significance for Human Health. Phytochem. Rev..

[20] Cartea M.E., Velasco P., Obregón S., Padilla G., De Haro A. (2008). Seasonal Variation in Glucosinolate Content in *Brassica oleracea* Crops Grown in Northwestern Spain. Phytochemistry.

[21] Velasco P., Cartea M.E., González C., Vilar M., Ordás A. (2007). Factors Affecting the Glucosinolate Content of Kale (*Brassica oleracea Acephala* Group). J. Agric. Food Chem..

[22] Galanakis C.M. (2019). Glucosinolates: Properties, Recovery, and Applications.

[23] Sikorska-Zimny K., Beneduce L. (2021). The Metabolism of Glucosinolates by Gut Microbiota. Nutrients.

[24] Del Carmen Martínez-Ballesta M., Moreno D., Carvajal M. (2013). The Physiological Importance of Glucosinolates on Plant Response to Abiotic Stress in *Brassica*. Int. J. Mol. Sci..

[25] Janczewski L. (2022). Sulforaphane and Its Bifunctional Analogs: Synthesis and Biological Activity. Molecules.

[26] Yagishita Y., Fahey J., Dinkova-Kostova A., Kensler T. (2019). Broccoli or Sulforaphane: Is It the Source or Dose that Matters?. Molecules.

[27] Kamal R., Razis A., Sukri N., Perimal E., Ahmad H., Patrick R., Djedaini-Pilard F., Mazzon E., Rigaud S. (2022). Beneficial Health Effects of Glucosinolates-Derived Isothiocyanates on Cardiovascular and Neurodegenerative Diseases. Molecules.

[28] Kuchernig J.C., Burow M., Wittstock U. (2012). Evolution of specifier proteins in glucosinolate-containing plants. BMC Evol. Biol..

[29] Baskar V., Park S.W., Nile S.H. (2016). An Update on Potential Perspectives of Glucosinolates on Protection against Microbial Pathogens and Endocrine Dysfunctions in Humans. Crit. Rev. Food Sci. Nutr..

[30] Brown A.F., Yousef G.G., Reid R.W., Chebrolu K.K., Thomas A., Krueger C., Jeffery E., Jackson E., Juvik J.A. (2015). Genetic Analysis of Glucosinolate Variability in Broccoli Florets Using Genome-Anchored Single Nucleotide Polymorphisms. Theor. Appl. Genet..

[31] Cieslik E., Leszczynska T., Filipiak-Florkiewicz A., Sikora E., Pisulewski P. (2007). Effects of Some Technological Processes on Glucosinolate Contents in Cruciferous Vegetables. Food Chem..

[32] Tarar A., Peng S., Cheema S., Peng C.-A. (2022). Anticancer Activity, Mechanism, and Delivery of Allyl Isothiocyanate. Bioengineering.

[33] Nakamura Y., Yoshimoto M., Murata Y., Shimoishi Y., Asai Y., Park E.Y., Sato K., Nakamura Y. (2007). Papaya Seed Represents a Rich Source of Biologically Active Isothiocyanate. J. Agric. Food Chem..

[34] Vicas S.I., Teusdea A.C., Carbunar M., Socaci S.A., Socaciu C. (2013). Glucosinolates Profile and Antioxidant Capacity of Romanian *Brassica* Vegetables Obtained by Organic and Conventional Agricultural Practices. Plant Foods Hum. Nutr..

[35] Rose P., Won Y.K., Ong C.N., Whiteman M. (2005). β-Phenylethyl and 8-Methylsulphinyloctyl Isothiocyanates, Constituents of Watercress, Suppress LPS Induced Production of Nitric Oxide and Prostaglandin E2 in RAW 264.7 Macrophages. Nitric Oxide.

[36] Justen V.L., Cohen J.D., Gardner G., Fritz V.A. (2011). Seasonal Variation in Glucosinolate Accumulation in Turnip Cultivars Grown with Colored Plastic Mulches. HortScience.

[37] Hanlon P.R., Barnes D.M. (2011). Phytochemical Composition and Biological Activity of 8 Varieties of Radish (*Raphanus sativus* L.) Sprouts and Mature Taproots. J. Food Sci..

[38] Possenti M., Baima S., Raffo A., Durazzo A., Giusti A.M., Natella F., Mérillon J.-M., Ramawat K.G. (2016). Glucosinolates in Food. Glucosinolates.

[39] Maldini M., Maksoud S.A., Natella F., Montoro P., Petretto G.L., Foddai M., De Nicola G.R., Chessa M., Pintore G. (2014). Moringa Oleifera: Study of Phenolics and Glucosinolates by Mass Spectrometry. J. Mass Spectrom..

[40] Clarke D. (2010). Glucosinolates, Structures and Analysis in Food. Anal. Methods.

[41] Juge N., Mithen R., Traka M. (2007). Molecular Basis for Chemoprevention by Sulforaphane: A Comprehensive Review. Cell. Mol. Life Sci..

[42] Orlando P., Nartea A., Silvestri S., Marcheggiani F., Cirilli I., Dludla P., Fiorini R., Pacetti D., Loizzo M., Lucci P. (2022). Bioavailability Study of Isothiocyanates and Other Bioactive Compounds of *Brassica oleracea* L. Var. Italica Boiled or Steamed: Functional Food or Dietary Supplement?. Antioxidants.

[43] Zhi Q., Li Y., Li F., Tian Y., Li F., Tang Y., Yang Y., Yin R., Ming J. (2018). Polyphenols Extracted from Coreopsis Tinctoria Buds Exhibited a Protective Effect against Acute Liver Damage. J. Funct. Foods.

[44] Lv Q., Li X., Fan B., Zhu C., Chen Z. (2022). The Cellular and Subcellular Organization of the Glucosinolate-Myrosinase System against Herbivores and Pathogens. Int. J. Mol. Sci..

[45] Prieto M., López C., Simal-Gandara J. (2019). Glucosinolates: Molecular Structure, Breakdown, Genetic, Bioavailability, Properties and Healthy and Adverse Effects. Functional Food Ingredients from Plants.

[46] Liou C.S., Sirk S.J., Diaz C.A., Klein A.P., Fischer C.R., Higginbottom S.K., Erez A., Donia M.S., Sonnenburg J.L., Sattely E.S. (2020). A metabolic pathway for activation of dietary glucosinolates by a human gut symbiont. Cell.

[47] Elfoul L., Rabot S., Khelifa N., Quinsac A., Duguay A., Rimbault A. (2001). Formation of allyl isothiocyanate from sinigrin in the digestive tract of rats monoassociated with a human colonic strain of Bacteroides thetaiotaomicron. FEMS Microbiol. Lett..

[48] Sivapalan T., Melchini A., Saha S., Needs P.W., Traka M.H., Tapp H., Dainty J.R., Mithen R.F. (2018). Bioavailability of glucoraphanin and sulforaphane from high-glucoraphanin broccoli. Mol. Nutr. Food Res..

[49] Li F., Hullar M., Beresford S., Lampe J. (2011). Variation of Glucoraphanin Metabolism in Vivo and Ex Vivo by Human Gut Bacteria. Br. J. Nutr..

[50] Clarke J.D., Dashwood R.H., Ho E. (2008). Multi-targeted prevention of cancer by sulforaphane. Cancer Lett..

[51] Winde I., Wittstock U. (2011). Insect Herbivore Counteradaptations to the Plant Glucosinolate–Myrosinase System. Phytochemistry.

[52] Barba F.J., Nikmaram N., Roohinejad S., Khelfa A., Zhu Z., Koubaa M. (2016). Bioavailability of glucosinolates and their breakdown products: Impact of processing. Front. Nutr..

[53] Rangkadilok N., Tomkins B., Nicolas M.E., Premier R.R., Bennett R.N., Eagling D.R., Taylor P.W.J. (2002). The Effect of Post-Harvest and Packaging Treatments on Glucoraphanin Concentration in Broccoli (*Brassica oleracea* Var. Italica). J. Agric. Food Chem..

[54] Badełek E., Kosson R., Adamicki F. (2012). The Effect of Storage in Controlled Atmosphere on the Quality and Health-Promoting Components of Broccoli (*Brassica oleracea* bar. Italica). J. Fruit Ornam. Plant Res..

[55] Tiwari U., Sheehy E., Rai D., Gaffney M., Evans P., Cummins E. (2015). Quantitative Human Exposure Model to Assess the Level of Glucosinolates upon Thermal Processing of Cruciferous Vegetables. LWT-Food Sci. Technol..

[56] Oliviero T., Verkerk R., Dekker M. (2018). Isothiocyanates from *Brassica* Vegetables Effects of Processing, Cooking, Mastication, and Digestion. Mol. Nutr. Food Res..

[57] Abdel-Massih R., Debs E., Othman L., Attieh J., Cabrerizo F. (2023). Glucosinolates, a Natural Chemical Arsenal: More to Tell than the Myrosinase Story. Front. Microbiol..

[58] Renz M., Andernach L., Kaufmann M., Rohn S., Hanschen F. (2023). Degradation of Glucosinolates and Formation of Isothiocyanates, Nitriles, Amines, and N,N′-dialk(en)yl thioureas during Domestic Boiling of Red Cabbage. Food Chem..

[59] Hwang E.-S., Kim G.-H. (2013). Effects of Various Heating Methods on Glucosinolate, Carotenoid and Tocopherol Concentrations in Broccoli. Int. J. Food Sci. Nutr..

[60] Wu W., Chen J., Yu D., Chen S., Ye X., Zhang Z. (2021). Analysis of Processing Effects on Glucosinolate Profiles in Red Cabbage by LC-MS/MS in Multiple Reaction Monitoring Mode. Molecules.

[61] Lafarga T., Bobo G., Viñas I., Collazo C., Aguiló-Aguayo I. (2018). Effects of Thermal and Non-Thermal Processing of Cruciferous Vegetables on Glucosinolates and Its Derived Forms. J. Food Sci. Technol..

[62] Ciska E., Honke J., Drabińska N. (2021). Changes in Glucosinolates and Their Breakdown Products during the Fermentation of Cabbage and Prolonged Storage of Sauerkraut: Focus on Sauerkraut Juice. Food Chem..

[63] Frandsen H., Markedal K., Martín-Belloso O., Sánchez-Vega R., Soliva-Fortuny R., Sorensen H., Sorensen S., Sorensen J. (2014). Effects of Novel Processing Techniques on Glucosinolates and Membrane Associated Myrosinases in Broccoli. Pol. J. Food Nutr. Sci..

[64] Westphal A., Riedl K.M., Cooperstone J.L., Kamat S., Balasubramaniam V.M., Schwartz S.J., Böhm V. (2017). High-Pressure Processing of Broccoli Sprouts: Influence on Bioactivation of Glucosinolates to Isothiocyanates. J. Agric. Food Chem..

[65] Baenas N., Marhuenda J., García-Viguera C., Moreno D.A., Ferreres F. (2019). Influence of Cooking Methods on Glucosinolates and Isothiocyanates Content in Novel Cruciferous Foods. Foods.

[66] Song L., Thornalley P.J. (2007). Effect of storage, processing and cooking on glucosinolate content of *Brassica* vegetables. Food Chem. Toxicol..

[67] Aires A., Carvalho R., Rosa E. (2012). Glucosinolate Composition of *Brassica* is Affected by Postharvest, Food Processing and Myrosinase Activity. J. Food Process. Preserv..

[68] Wang Z., Kwan M.L., Pratt R., Tang L. (2020). Effects of cooking methods on total isothiocyanate yield from cruciferous vegetables. Food Sci. Nutr..

[69] Verkerk R., Dekker M. (2004). Glucosinolates and Myrosinase Activity in Red Cabbage (*Brassica oleracea* L. Var. *Capitata* f. *rubra* DC.) after Various Microwave Treatments. J. Agric. Food Chem..

[70] Nandasiri R., Semenko B., Wijekoon C., Suh M. (2023). Air-Frying Is a Better Thermal Processing Choice for Improving Antioxidant Properties of *Brassica* Vegetables. Antioxidants.

[71] Vallejo F., Tomás-Barberán F., García-Viguera C. (2002). Glucosinolates and Vitamin C Content in Edible Parts of Broccoli Florets after Domestic Cooking. Eur. Food Res. Technol..

[72] Miękus N., Marszałek K., Podlacha M., Iqbal A., Puchalski C., Świergiel A.H. (2020). Health Benefits of Plant-Derived Sulfur Compounds, Glucosinolates, and Organosulfur Compounds. Molecules.

[73] Manchali S., Chidambara Murthy K.N., Patil B.S. (2012). Crucial Facts about Health Benefits of Popular Cruciferous Vegetables. J. Funct. Foods.

[74] Lund E. (2003). Non-Nutritive Bioactive Constituents of Plants: Dietary Sources and Health Benefits of Glucosinolates. Int. J. Vitam. Nutr. Res..

[75] Johnson I. (2018). Cruciferous Vegetables and Risk of Cancers of the Gastrointestinal Tract. Mol. Nutr. Food Res..

[76] Connolly E., Liu A.H., Radavelli-Bagatini S., Shafaei A., Boyce M.C., Wood L.G., McCahon L., Koch H., Sim M., Hill C.R. (2024). Cruciferous Vegetables Lower Blood Pressure in Adults with Mildly Elevated Blood Pressure in a Randomized, Controlled, Crossover Trial: The VEgetableS for vaScular hEaLth (VESSEL) Study. BMC Med..

[77] Abellán A., Domínguez-Perles R., Moreno D., García-Viguera C. (2019). Sorting out the Value of Cruciferous Sprouts as Sources of Bioactive Compounds for Nutrition and Health. Nutrients.

[78] Sturm C., Wagner A. (2017). *Brassica*-Derived Plant Bioactives as Modulators of Chemopreventive and Inflammatory Signaling Pathways. Int. J. Mol. Sci..

[79] Clarke J., Hsu A., Riedl K., Bella D., Schwartz S., Stevens J., Ho E. (2011). Bioavailability and Inter-Conversion of Sulforaphane and Erucin in Human Subjects Consuming Broccoli Sprouts or Broccoli Supplement in a Cross-over Study Design. Pharmacol. Res..

[80] Armah C., Traka M., Dainty J., Defernez M., Janssens A., Leung W., Doleman J., Potter J., Mithen R. (2013). A Diet Rich in High-Glucoraphanin Broccoli Interacts with Genotype to Reduce Discordance in Plasma Metabolite Profiles by Modulating Mitochondrial Function. Am. J. Clin. Nutr..

[81] Keum Y., Jeong W., Kong A. (2004). Chemoprevention by Isothiocyanates and Their Underlying Molecular Signaling Mechanisms. Mutat. Res.-Fundam. Mol. Mech. Mutagen..

[82] Murashima M., Watanabe S., Zhuo X., Uehara M., Kurashige A. (2004). Phase 1 Study of Multiple Biomarkers for Metabolism and Oxidative Stress after One-Week Intake of Broccoli Sprouts. BioFactors.

[83] Christiansen B., Muguerza N., Petersen A., Kveiborg B., Madsen C., Thomas H., Ihlemann N., Sorensen J., Kober L., Sorensen H. (2010). Ingestion of Broccoli Sprouts Does Not Improve Endothelial Function in Humans with Hypertension. PLoS ONE.

[84] Bahadoran Z., Mirmiran P., Hosseinpanah F., Hedayati M., Hosseinpour-Niazi S., Azizi F. (2011). Broccoli Sprouts Reduce Oxidative Stress in Type 2 Diabetes: A Randomized Double-Blind Clinical Trial. Eur. J. Clin. Nutr..

[85] Bahadoran Z., Mirmiran P., Hosseinpanah F., Rajab A., Asghari G., Azizi F. (2012). Broccoli Sprouts Powder Could Improve Serum Triglyceride and Oxidized LDL/LDL-Cholesterol Ratio in Type 2 Diabetic Patients: A Randomized Double-Blind Placebo-Controlled Clinical Trial. Diabetes Res. Clin. Pract..

[86] Armah C., Derdemezis C., Traka M., Dainty J., Doleman J., Saha S., Leung W., Potter J., Lovegrove J., Mithen R. (2015). Diet Rich in High Glucoraphanin Broccoli Reduces Plasma LDL Cholesterol: Evidence from Randomised Controlled Trials. Mol. Nutr. Food Res..

[87] López-Chillón M., Carazo-Díaz C., Prieto-Merino D., Zafrilla P., Moreno D., Villaño D. (2019). Effects of Long-Term Consumption of Broccoli Sprouts on Inflammatory Markers in Overweight Subjects. Clin. Nutr..

[88] Langston-Cox A., Anderson D., Creek D., Palmer K., Marshall S., Wallace E. (2021). Sulforaphane Bioavailability and Effects on Blood Pressure in Women with Pregnancy Hypertension. Reprod. Sci..

[89] Na G., He C., Zhang S., Tian S., Bao Y., Shan Y. (2023). Dietary Isothiocyanates: Novel Insights into the Potential for Cancer Prevention and Therapy. Int. J. Mol. Sci..

[90] Wojtasinska A., Kucmierz J., Tokarek J., Dybiec J., Rodzen A., Mlynarska E., Rysz J., Franczyk B. (2023). New Insights into Cardiovascular Diseases Treatment Based on Molecular Targets. Int. J. Mol. Sci..

[91] Bai Y., Wang X., Zhao S., Ma C., Cui J., Zheng Y. (2015). Sulforaphane Protects against Cardiovascular Disease via Nrf2 Activation. Oxidative Med. Cell. Longev..

[92] Shah S., Akram M., Riaz M., Munir N., Rasool G. (2019). Cardioprotective Potential of Plant-Derived Molecules: A Scientific and Medicinal Approach. Dose-Response.

[93] Connolly E., Sim M., Travica N., Marx W., Beasy G., Lynch G., Bondonno C., Lewis J., Hodgson J., Blekkenhorst L. (2021). Glucosinolates from Cruciferous Vegetables and Their Potential Role in Chronic Disease: Investigating the Preclinical and Clinical Evidence. Front. Pharmacol..

[94] Kala C., Ali S., Ahmad N., Gilani S., Khan N. (2018). Isothiocyanates: A Review. Res. J. Pharmacogn..

[95] Dinkova-Kostova A., Kostov R. (2012). Glucosinolates and Isothiocyanates in Health and Disease. Trends Mol. Med..

[96] Corssac G., Campos-Carraro C., Hickmann A., Araujo A., Fernandes R., Belló-Klein A. (2018). Sulforaphane Effects on Oxidative Stress Parameters in Culture of Adult Cardiomyocytes. Biomed. Pharmacother..

[97] Angeloni C., Hrelia S., Malaguti M. (2017). Neuroprotective Effects of Glucosinolates. Glucosinolates.

[98] Fuentes F., Paredes-Gonzalez X., Kong A.N. (2015). Dietary Glucosinolates Sulforaphane, Phenethyl Isothiocyanate, Indole-3-Carbinol/3,3′-Diindolylmethane: Anti-Oxidative Stress/Inflammation, Nrf2, Epigenetics/Epigenomics and In Vivo Cancer Chemopreventive Efficacy. Curr. Pharmacol. Rep..

[99] Calmes B., N’Guyen G., Dumur J., Brisach C., Campion C., Iacomi B., Pigné S., Dias E., Macherel D., Guillemette T. (2015). Glucosinolate-Derived Isothiocyanates Impact Mitochondrial Function in Fungal Cells and Elicit an Oxidative Stress Response Necessary for Growth Recovery. Front. Plant Sci..

[100] Mohammed E., El-Naga R., Lotfy R., Al-Gendy A., El-Demerdash E. (2017). Anti-Fibrotic Potential of a Matthiola Arabica Isothiocyanates Rich Fraction: Impact on Oxidative Stress, Inflammatory and Fibrosis Markers. Die Pharm..

[101] Clemente M., Miguel M., Felipe K., Gribner C., Moura P., Rigoni A., Parisotto E., Piltz M., Valdameri G., Henneberg R. (2020). Biomarkers of Oxidative Stress and Inflammation in People Witha Physical Disability Treated with a Standardized Extract of Nasturtium Officinale: A Randomized, Double-Blind, and Placebo-Controlled Trial. Phytother. Res..

[102] Zhou Y., Xu X., Wu J., Xu L., Zhang M., Li Z., Wang D. (2020). Allyl Isothiocyanate Treatment Alleviates Chronic Obstructive Pulmonary Disease through the Nrf2-Notch1 Signaling and Upregulation of MRP1. Life Sci..

[103] Lee Y., Seon M., Cho H., Kim J., Park J. (2009). Benzyl Isothiocyanate Exhibits Anti-Inflammatory Effects in Murine Macrophages and in Mouse Skin. J. Mol. Med..

[104] Huang C., Lin A., Liu C., Tsai C., Chang I., Chen H., Lii C. (2013). Isothiocyanates Protect against Oxidized LDL-Induced Endothelial Dysfunction by Upregulating Nrf2-Dependent Antioxidation and Suppressing NFκB Activation. Mol. Nutr. Food Res..

[105] Mahn A., Castillo A. (2021). Potential of Sulforaphane as a Natural Immune System Enhancer: A Review. Molecules.

[106] Patel V., Dial K., Wu J., Gauthier A., Wu W., Lin M., Espey M., Thomas D., Ashby C., Mantell L. (2020). Dietary Antioxidants Significantly Attenuate Hyperoxia-Induced Acute Inflammatory Lung Injury by Enhancing Macrophage Function via Reducing the Accumulation of Airway HMGB1. Int. J. Mol. Sci..

[107] Chen X., Dodd G., Kunsch C. (2009). Sulforaphane Inhibits TNF-α-Induced Activation of P38 MAP Kinase and VCAM-1 and MCP-1 Expression in Endothelial Cells. Inflamm. Res..

[108] Liu Y., Hsieh C., Weng Y., Chuang S., Hsieh C., Wung B. (2008). Sulforaphane Inhibition of Monocyte Adhesion via the Suppression of ICAM-1 and NF-κB Is Dependent upon Glutathione Depletion in Endothelial Cells. Vasc. Pharmacol..

[109] Hung C., Huang H., Wang C., Liu K., Lii C. (2014). Sulforaphane Inhibits TNF-α-Induced Adhesion Molecule Expression Through the Rho A/ROCK/NF-κB Signaling Pathway. J. Med. Food.

[110] Silva-Palacios A., Ostolga-Chavarría M., Sánchez-Garibay C., Rojas-Morales P., Galván-Arzate S., Buelna-Chontal M., Pavón N., Pedraza-Chaverrí J., Königsberg M., Zazueta C. (2019). Sulforaphane Protects from Myocardial Ischemia-Reperfusion Damage through the Balanced Activation of Nrf2/AhR. Free Radic. Biol. Med..

[111] Bai Y., Chen Q., Sun Y., Wang X., Lv L., Zhang L., Liu J., Zhao S., Wang X. (2017). Sulforaphane Protection against the Development of Doxorubicin-Induced Chronic Heart Failure Is Associated with Nrf2 Upregulation. Cardiovasc. Ther..

[112] Kee H., Kim G., Kim I., Jeong M. (2015). Sulforaphane Suppresses Cardiac Hypertrophy by Inhibiting GATA4/GATA6 Expression and MAPK Signaling Pathways. Mol. Nutr. Food Res..

[113] Ma T., Zhu D., Chen D., Zhang Q., Dong H., Wu W., Lu H., Wu G. (2018). Sulforaphane, a Natural Isothiocyanate Compound, Improves Cardiac Function and Remodeling by Inhibiting Oxidative Stress and Inflammation in a Rabbit Model of Chronic Heart Failure. Med. Sci. Monit..

[114] Martelli A., Piragine E., Citi V., Testai L., Pagnotta E., Ugolini L., Lazzeri L., Di Cesare Mannelli L., Manzo O.L., Bucci M. (2020). Erucin Exhibits Vasorelaxing Effects and Antihypertensive Activity by H2 S-releasing Properties. Br. J. Pharmacol..

[115] Gladine C., Combe N., Vaysse C., Pereira B., Huertas A., Salvati S., Rossignol-Castera A., Cano N., Chardigny J. (2013). Optimized Rapeseed Oil Enriched with Healthy Micronutrients: A Relevant Nutritional Approach to Prevent Cardiovascular Diseases. Results of the Optim’Oils Randomized Intervention Trial. J. Nutr. Biochem..

[116] Heggen E., Granlund L., Pedersen J., Holme I., Ceglarek U., Thiery J., Kirkhus B., Tonstad S. (2010). Plant Sterols from Rapeseed and Tall Oils: Effects on Lipids, Fat-Soluble Vitamins and Plant Sterol Concentrations. Nutr. Metab. Cardiovasc. Dis..

[117] Le T., Chiu C., Hsieh P. (2020). Bioactive Compounds and Bioactivities of *Brassica oleracea* L. Var. Italica Sprouts and Microgreens: An. Updated Overview from a Nutraceutical Perspective. Plants.

[118] Mordecai J., Ullah S., Ahmad I. (2023). Sulforaphane and Its Protective Role in Prostate Cancer: A Mechanistic Approach. Int. J. Mol. Sci..

[119] Gu H., Mao X., Du M. (2020). Metabolism, Absorption, and Anti-Cancer Effects of Sulforaphane: An Update. Crit. Rev. Food Sci. Nutr..

[120] Núñez-Iglesias M.J., Novío S., García C., Pérez-Muñuzuri E., Soengas P., Cartea E., Velasco P., Freire-Garabal M. (2019). Glucosinolate-degradation products as co-adjuvant therapy on prostate cancer in vitro. Int. J. Mol. Sci..

[121] Singh K., Hahm E., Alumkal J., Foley L., Hitchens T., Shiva S., Parikh R., Jacobs B., Singh S. (2019). Reversal of the Warburg Phenomenon in Chemoprevention of Prostate Cancer by Sulforaphane. Carcinogenesis.

[122] Ahmed Z., Li X., Li F., Cheaito H., Patel K., Mosallam E., Elbargeesy G., Dou Q. (2018). Computational and Biochemical Studies of Isothiocyanates as Inhibitors of Proteasomal Cysteine Deubiquitinases in Human Cancer Cells. J. Cell. Biochem..

[123] Rutz J., Thaler S., Maxeiner S., Chun F., Blaheta R. (2020). Sulforaphane Reduces Prostate Cancer Cell Growth and Proliferation In Vitro by Modulating the Cdk-Cyclin Axis and Expression of the CD44 Variants 4, 5, and 7. Int. J. Mol. Sci..

[124] Singh K., Kim S., Hahm E., Pore S., Jacobs B., Singh S. (2018). Prostate Cancer Chemoprevention by Sulforaphane in a Preclinical Mouse Model Is Associated with Inhibition of Fatty Acid Metabolism. Carcinogenesis.

[125] Zhang C., Su Z., Khor T., Shu L., Kong A. (2013). Sulforaphane Enhances Nrf2 Expression in Prostate Cancer TRAMP C1 Cells through Epigenetic Regulation. Biochem. Pharmacol..

[126] Negele L., Schneider B., Ristl R., Stulnig T., Willfort-Ehringer A., Helk O., Widhalm K. (2015). Effect of a Low-Fat Diet Enriched Either with Rapeseed Oil or Sunflower Oil on Plasma Lipoproteins in Children and Adolescents with Familial Hypercholesterolaemia. Results of a Pilot Study. Eur. J. Clin. Nutr..

[127] Baldelli S., Aquilano K., Ciriolo M. (2013). Punctum on Two Different Transcription Factors Regulated by PGC-1α: Nuclear Factor Erythroid-Derived 2-like 2 and Nuclear Respiratory Factor 2. Biochim. Biophys. Acta-Gen. Subj..

[128] Ali M., Khan N., Kaleem N., Ahmad W., Alharethi S., Alharbi B., Alhassan H., Al-Enazi M., Razis A., Modu B. (2023). Anticancer Properties of Sulforaphane: Current Insights at the Molecular Level. Front. Oncol..

[129] Traka M., Melchini A., Coode-Bate J., Al Kadhi O., Saha S., Defernez M., Troncoso-Rey P., Kibblewhite H., O’Neill C., Bernuzzi F. (2019). Transcriptional Changes in Prostate of Men on Active Surveillance after a 12-Mo Glucoraphanin-Rich Broccoli Intervention-Results from the Effect of Sulforaphane on Prostate Cancer Prevention (ESCAPE) Randomized Controlled Trial. Am. J. Clin. Nutr..

[130] Zhang Z., Garzotto M., Davis E., Mori M., Stoller W., Farris P., Wong C., Beaver L., Thomas G., Williams D.E. (2019). Sulforaphane Bioavailability and Chemopreventive Activity in Men Presenting for Biopsy of the Prostate Gland: A Randomized Controlled Trial. Nutr. Cancer-Int. J..

[131] Zhang F., Wan X., Zhan J., Shen M., Li R. (2024). Sulforaphane Inhibits the Growth of Prostate Cancer by Regulating the microRNA-3919/DJ-1 Axis. Front. Oncol..

[132] Lewinska A., Adamczyk-Grochala J., Deregowska A., Wnuk M. (2017). Sulforaphane-Induced Cell Cycle Arrest and Senescence Are Accompanied by DNA Hypomethylation and Changes in microRNA Profile in Breast Cancer Cells. Theranostics.

[133] Jackson S., Singletary K. (2004). Sulforaphane: A Naturally Occurring Mammary Carcinoma Mitotic Inhibitor, Which Disrupts Tubulin Polymerization. Carcinogenesis.

[134] Jackson S., Singletary K. (2004). Sulforaphane Inhibits Human MCF-7 Mammary Cancer Cell Mitotic Progression and Tubulin Polymerization. J. Nutr..

[135] Alhazmi N., Pai C., Albaqami A., Wang H., Zhao X., Chen M., Hu P., Guo S., Starost K., Hajihassani O. (2020). The Promyelocytic Leukemia Protein Isoform PML1 Is an Oncoprotein and a Direct Target of the Antioxidant Sulforaphane (SFN). Biochim. Biophys. Acta-Mol. Cell Res..

[136] Pawlik A., Slominska-Wojewódzka M., Herman-Antosiewicz A. (2016). Sensitization of Estrogen Receptor-Positive Breast Cancer Cell Lines to 4-Hydroxytamoxifen by Isothiocyanates Present in Cruciferous Plants. Eur. J. Nutr..

[137] Royston K., Udayakumar N., Lewis K., Tollefsbol T. (2017). A Novel Combination of Withaferin A and Sulforaphane Inhibits Epigenetic Machinery, Cellular Viability and Induces Apoptosis of Breast Cancer Cells. Int. J. Mol. Sci..

[138] Hussain A., Mohsin J., Prabhu S., Begum S., Nusri Q., Harish G., Javed E., Khan M., Sharma C. (2013). Sulforaphane Inhibits Growth of Human Breast Cancer Cells and Augments the Therapeutic Index of the Chemotherapeutic Drug, Gemcitabine. Asian Pac. J. Cancer Prev..

[139] Sarkar R., Mukherjee S., Biswas J., Roy M. (2012). Sulphoraphane, a Naturally Occurring Isothiocyanate Induces Apoptosis in Breast Cancer Cells by Targeting Heat Shock Proteins. Biochem. Biophys. Res. Commun..

[140] Kanematsu S., Uehara N., Miki H., Yoshizawa K., Kawanaka A., Yuri T., Tsubura A. (2010). Autophagy Inhibition Enhances Sulforaphane-Induced Apoptosis in Human Breast Cancer Cells. Anticancer Res..

[141] Bagheri M., Fazli M., Saeednia S., Kharanagh M., Ahmadiankia N. (2020). Sulforaphane Modulates Cell Migration and Expression of β-Catenin and Epithelial Mesenchymal Transition Markers in Breast Cancer Cells. Iran. J. Public Health.

[142] Atwell L., Zhang Z., Mori M., Farris P., Vetto J., Naik A., Oh K., Thuillier P., Ho E., Shannon J. (2015). Sulforaphane Bioavailability and Chemopreventive Activity in Women Scheduled for Breast Biopsy. Cancer Prev. Res..

[143] Zhang Z., Atwell L., Farris P., Ho E., Shannon J. (2016). Associations between Cruciferous Vegetable Intake and Selected Biomarkers among Women Scheduled for Breast Biopsies. Public Health Nutr..

[144] Yan L., Yan Y. (2023). Therapeutic Potential of Sulforaphane in Liver Diseases: A Review. Front. Pharmacol..

[145] Liu P., Atkinson S., Akbareian S., Zhou Z., Munsterberg A., Robinson S., Bao Y. (2017). Sulforaphane Exerts Anti-Angiogenesis Effects against Hepatocellular Carcinoma through Inhibition of STAT3/HIF-1α/VEGF Signalling. Sci. Rep..

[146] Zou X., Qu Z., Fang Y., Shi X., Ji Y. (2017). Endoplasmic Reticulum Stress Mediates Sulforaphane-Induced Apoptosis of HepG2 Human Hepatocellular Carcinoma Cells. Mol. Med. Rep..

[147] Moon D., Kang S., Kim K., Kim M., Choi Y., Kim G. (2010). Sulforaphane Decreases Viability and Telomerase Activity in Hepatocellular Carcinoma Hep3B Cells through the Reactive Oxygen Species-Dependent Pathway. Cancer Lett..

[148] Wu J., Han J., Hou B., Deng C., Wu H., Shen L. (2016). Sulforaphane Inhibits TGF-β-Induced Epithelial-Mesenchymal Transition of Hepatocellular Carcinoma Cells via the Reactive Oxygen Species-Dependent Pathway. Oncol. Rep..

[149] Chen Y., Wallig M., Jeffery E. (2016). Dietary Broccoli Lessens Development of Fatty Liver and Liver Cancer in Mice Given Diethylnitrosamine and Fed a Western or Control Diet. J. Nutr..

[150] Rudolf E., Cervinka M. (2011). Sulforaphane Induces Cytotoxicity and Lysosome- and Mitochondria-Dependent Cell Death in Colon Cancer Cells with Deleted P53. Toxicol. Vitr..

[151] Liu K., Shih T., Kuo C., Ma Y., Yang J., Wu P., Huang Y., Lai K., Chung J. (2016). Sulforaphane Induces Cell Death Through G2/M Phase Arrest and Triggers Apoptosis in HCT 116 Human Colon Cancer Cells. Am. J. Chin. Med..

[152] Chen Y., Wang M., Zhu J., Xie C., Li X., Wu J., Geng S., Han H., Zhong C. (2020). TAp63α Targeting of Lgr5 Mediates Colorectal Cancer Stem Cell Properties and Sulforaphane Inhibition. Oncogenesis.

[153] Coutinho L., Tortelli T.J., Rangel M. (2023). Sulforaphane: An Emergent Anti-Cancer Stem Cell Agent. Front. Oncol..

[154] Nambiar D.M., Kumari J., Augustine R., Kumar P., Bajpai P.K., Bisht N.C. (2021). GTR1 and GTR2 transporters differentially regulate tissue-specific glucosinolate contents and defence responses in the oilseed crop *Brassica juncea*. Plant Cell Environ..

[155] Xu D., Sanden N.C.H., Hansen L.L., Belew Z.M., Madsen S.R., Meyer L., Jørgensen M.E., Hunziker P., Veres D., Crocoll C. (2023). Export of defensive glucosinolates is key for their accumulation in seeds. Nature.

[156] Kapusta-Duch J., Kusznierewicz B., Leszczyńska T., Borczak B. (2016). Effect of cooking on the contents of glucosinolates and their degradation products in selected *Brassica* vegetables. J. Funct. Foods.

[157] Hossain A., Skalicky M., Brestic M., Maitra S., Sarkar S., Ahmad Z., Vemuri H., Garai S., Mondal M., Bhatt R. (2021). Selenium Biofortification: Roles, Mechanisms, Responses and Prospects. Molecules.

[158] Bouranis D., Stylianidis G., Manta V., Karousis E., Tzanaki A., Dimitriadi D., Bouzas E., Siyiannis V., Constantinou-Kokotou V., Chorianopoulou S.N. (2023). Floret Biofortification of Broccoli Using Amino Acids Coupled with Selenium under Different Surfactants: A Case Study of Cultivating Functional Foods. Plants.

[159] Schiavon M., Berto C., Malagoli M., Trentin A., Sambo P., Dall’Acqua S., Pilon-Smits E. (2016). Selenium Biofortification in Radish Enhances Nutritional Quality via Accumulation of Methyl-Selenocysteine and Promotion of Transcripts and Metabolites Related to Glucosinolates, Phenolics, and Amino Acids. Front. Plant Sci..

[160] Guardiola-Márquez C., García-Sánchez C., Sánchez-Arellano O., Bojorquez-Rodríguez E., Jacobo-Velázquez D. (2023). Biofortification of Broccoli Microgreens (*Brassica oleracea* Var. Italica) with Glucosinolates, Zinc, and Iron through the Combined Application of Bio- and Nanofertilizers. Foods.

[161] Barrameda-Medina Y., Blasco B., Lentini M., Esposito S., Baenas N., Moreno D., Ruiz J. (2017). Zinc Biofortification Improves Phytochemicals and Amino-Acidic Profile in *Brassica oleracea* Cv. Bronco. Plant Sci..

[162] Ramchiary N., Bisht N., Gupta V., Mukhopadhyay A., Arumugam N., Sodhi Y., Pental D., Pradhan A. (2007). QTL Analysis Reveals Context-Dependent Loci for Seed Glucosinolate Trait in the Oilseed *Brassica juncea*: Importance of Recurrent Selection Backcross Scheme for the Identification of “true” QTL. Theor. Appl. Genet..

[163] Neequaye M., Steuernagel B., Saha S., Trick M., Troncoso-Rey P., van den Bosch F., Traka M., Ostergaard L., Mithen R. (2022). Characterisation of the Introgression of *Brassica villosa* Genome into Broccoli to Enhance Methionine-Derived Glucosinolates and Associated Health Benefits. Front. Plant Sci..

[164] Yi G., Robin A., Yang K., Park J., Hwang B., Nou I. (2016). Exogenous Methyl Jasmonate and Salicylic Acid Induce Subspecies-Specific Patterns of Glucosinolate Accumulation and Gene Expression in *Brassica oleracea* L. Molecules.

[165] Qin H., King G., Borpatragohain P., Zou J. (2023). Developing Multifunctional Crops by Engineering Brassicaceae Glucosinolate Pathways. Plant Commun..

[166] Sohn S., Thamilarasan S., Pandian S., Oh Y., Ryu T., Lee G., Shin E. (2022). Interspecific Hybridization of Transgenic *Brassica napus* and *Brassica rapa*-An Overview. Genes.

[167] Patel A., Miles A., Strackhouse T., Cook L., Leng S., Patel S., Klinger K., Rudrabhatla S., Potlakayala S. (2023). Methods of Crop Improvement and Applications towards Fortifying Food Security. Front. Genome Ed..

[168] Nour-Eldin H.H., Andersen T.G., Burow M., Madsen S.R., Jørgensen M.E., Olsen C.E., Dreyer I., Hedrich R., Geiger D., Halkier B.A. (2012). NRT/PTR Transporters Are Essential for Translocation of Glucosinolate Defence Compounds to Seeds. Nature.

[169] Sánchez-Pujante P., Sabater-Jara A., Belchí-Navarro S., Pedreño M., Almagro L. (2019). Increased Glucosinolate Production in *Brassica oleracea* Var. Italica Cell Cultures Due to Coronatine Activated Genes Involved in Glucosinolate Biosynthesis. J. Agric. Food Chem..

[170] Qian H., Sun B., Miao H., Cai C., Xu C., Wang Q. (2015). Variation of Glucosinolates and Quinone Reductase Activity among Different Varieties of Chinese Kale and Improvement of Glucoraphanin by Metabolic Engineering. Food Chem..

[171] Miao H., Zeng W., Wang J., Zhang F., Sun B., Wang Q. (2021). Improvement of Glucosinolates by Metabolic Engineering in *Brassica* Crops. Abiotech.

[172] Bhaswant M., Shanmugam D., Miyazawa T., Abe C., Miyazawa T. (2023). Microgreens-A Comprehensive Review of Bioactive Molecules and Health Benefits. Molecules.

[173] Ebert A. (2022). Sprouts and Microgreens-Novel Food Sources for Healthy Diets. Plants.

[174] Sun J., Kou L., Geng P., Huang H., Yang T., Luo Y., Chen P. (2015). Metabolomic Assessment Reveals an Elevated Level of Glucosinolate Content in CaCl_2_ Treated Broccoli Microgreens. J. Agric. Food Chem..

[175] Lu Y., Dong W., Yang T., Luo Y., Chen P. (2021). Preharvest UVB Application Increases Glucosinolate Contents and Enhances Postharvest Quality of Broccoli Microgreens. Molecules.

[176] Hu Y., Li X., He X., He R., Li Y., Liu X., Liu H. (2022). Effects of Pre-Harvest Supplemental UV-A Light on Growth and Quality of Chinese Kale. Molecules.

[177] Sathasivam R., Park S., Kim J., Park Y., Kim M., Nguyen B., Lee S. (2023). Metabolic Profiling of Primary and Secondary Metabolites in Kohlrabi (*Brassica oleracea* Var. *gongylodes*) Sprouts Exposed to Different Light-Emitting Diodes. Plants.

[178] Zhao J., Zhang X., Li F., Lei X., Ge L., Li H., Zhao N., Ming J. (2024). The Effects of Interventions with Glucosinolates and Their Metabolites in Cruciferous Vegetables on Inflammatory Bowel Disease: A Review. Foods.

[179] Zhang Y., Zhang W., Zhao Y., Peng R., Zhang Z., Xu Z., Simal-Gandara J., Yang H., Deng J. (2024). Bioactive Sulforaphane from Cruciferous Vegetables: Advances in Biosynthesis, Metabolism, Bioavailability, Delivery, Health Benefits, and Applications. Crit. Rev. Food Sci. Nutr..

[180] Sharma S., Rani H., Kaur G., Kumar S., Sheikh S., Samota M.K. (2024). Comprehensive Overview of Glucosinolates in Crucifers: Occurrence, Roles, Metabolism, and Transport Mechanisms—A Review. Phytochem. Rev..

